# Branched chain amino acids attenuate major pathologies in mouse models of retinal degeneration and glaucoma

**DOI:** 10.1016/j.heliyon.2018.e00544

**Published:** 2018-03-01

**Authors:** Tomoko Hasegawa, Hanako Ohashi Ikeda, Sachiko Iwai, Yuki Muraoka, Tatsuaki Tsuruyama, Keiko Okamoto-Furuta, Haruyasu Kohda, Akira Kakizuka, Nagahisa Yoshimura

**Affiliations:** a Department of Ophthalmology and Visual Sciences, Kyoto University Graduate School of Medicine, Sakyo-ku, Kyoto, 606-8507, Japan; b Department of Experimental Therapeutics, Institute for Advancement of Clinical and Translational Science, Kyoto University Hospital, Sakyo-ku, Kyoto, 606-8507, Japan; c Center for Anatomical Studies, Kyoto University Graduate School of Medicine, Sakyo-ku, Kyoto, 606-8501, Japan; d Laboratory of Functional Biology, Kyoto University Graduate School of Biostudies, Sakyo-ku, Kyoto, 606-8501, Japan

**Keywords:** Ophthalmology

## Abstract

Retinal neuronal cell death underlies many incurable eye diseases such as retinitis pigmentosa (RP) and glaucoma, and causes adult blindness. We have shown that maintenance of ATP levels via inhibiting ATP consumption is a promising strategy for preventing neuronal cell death. Here, we show that branched chain amino acids (BCAAs) are able to increase ATP production by enhancing glycolysis. In cell culture, supplementation of the culture media with BCAAs, but not glucose alone, enhanced cellular ATP levels, which was canceled by a glycolysis inhibitor. Administration of BCAAs to RP mouse models, *rd10* and *rd12*, significantly attenuated photoreceptor cell death morphologically and functionally, even when administration was started at later stages. Administration of BCAAs in a glaucoma mouse model also showed significant attenuation of retinal ganglion cell death. These results suggest that administration of BCAAs could contribute to a comprehensive therapeutic strategy for retinal neurodegenerative diseases such as RP and glaucoma.

## 1 Introduction

Retinitis pigmentosa (RP) is one of several incurable eye diseases; it leads to blindness, with approximately 1.5 million people affected worldwide, and its incidence is about 1 in 4,000 people (Hartong et al., 2006). No established therapies are available, although potential therapies, including regenerative medicine (Ng et al., 2014), gene therapy (Acland et al., 2001), and neurotropic factor therapy (Sieving et al., 2006), are currently being pursued. A much more common disease, glaucoma, is one of the leading causes of blindness worldwide, accounting for 4.0–15.5% of adult blindness (Bourne et al., 2013), and it ranks in the top third of causes of incurable visual impairment in Western countries (Klaver et al., 1998); moreover, the number of patients is increasing world-wide (Quigley and Broman, 2006). Strategies to reduce intraocular pressure are used in glaucoma treatment (Collaborative Normal-Tension Glaucoma Study Group, 1998; Leske et al., 2004). However, there remain considerable numbers of patients, up to 33% of the total, whose visual field impairment progresses despite intraocular pressure within the normal limit (Collaborative Normal-Tension Glaucoma Study Group, 1998; Hitchings, 1992). Thus, new therapeutic strategies that prevent cell death and prevent or retard disease progression are eagerly awaited.

We previously focused our efforts on the maintenance of ATP levels by inhibiting ATP consumption. KUSs (Kyoto University Substances), which specifically block the ATPase activities of valosin-containing protein (VCP), the most abundant soluble ATPase in all types of cells including neurons, have shown promise for preventing neuronal cell death in mouse models of RP (Hasegawa et al., 2016b; Ikeda et al., 2014), glaucoma (Nakano et al., 2016), and retinal artery occlusion (Hata et al., 2017). The efficacies were apparently coupled with the mitigation of endoplasmic reticulum (ER) stress. Following this line of reasoning, an alternative, but not mutually exclusive, therapeutic strategy for the maintenance of ATP levels in the diseased states would be the enhancement of ATP production by novel compound(s).

It is known that neural cells, including retinal neurons, demand a large amount of energy (Futterman and Kinoshita, 1959; Winkler, 1981). Consistently, chronic hypoglycemia has been shown to be associated with retinal cell death in mice (Punzo et al., 2009; Umino et al., 2006). On the other hand, chronic hyperglycemia is related to diseased conditions such as diabetes mellitus, coronary heart disease, stroke, renal failure, and diabetic retinopathy (Zimmet et al., 2016). An epidemiological survey has shown that the incidence of glaucoma is high in patients suffering from diabetes mellitus (Zhao et al., 2015). Because ATP levels are a hallmark of cell health, these results indicate that excessive blood glucose is inefficiently used as a source of ATP, or is even harmful to cells in these pathological conditions. Nevertheless, glucose is the most abundant energy source in humans, especially those with high serum glucose. Thus, in order to enhance ATP production, it would be ideal to take advantage of high serum glucose by finessing metabolic regulatory pathways.

Branched chain amino acids (BCAAs) have aliphatic side-chains with branches, which include leucine (Leu), isoleucine (Ile), and valine (Val). Supplementation with BCAAs is commonly used by athletes to increase bulk and power of muscles, and thus it appears to counteract ATP depletion and possible muscle cell death, especially after strenuous training. Supplementation with BCAAs has also been used to improve hypoalbuminemia in patients with decompensated liver cirrhosis (Kawaguchi et al., 2014; Plauth et al., 2006). Efficacies of BCAAs in these patients have been evidenced by a reduced incidence of complications, such as liver cancer, rupture of esophageal varices, or progression to hepatic failure (Kawaguchi et al., 2014; Muto et al., 2005), indicating that BCAA administration is able to maintain or improve the functions of hepatic cells. BCAAs also activate mammalian target of rapamycin (mTOR) (Anthony et al., 2000; Efeyan et al., 2012; Matsumura et al., 2005; Proud, 2004a, 2004b), which regulates cell growth, proliferation, and survival (Hung et al., 2012; Yang and Guan, 2007). mTOR is activated by phosphorylation of Ser2481 and Ser2448 (Copp et al., 2009; Yang and Guan, 2007), and mTOR signaling is stimulated by amino acids, hormones, and mitogens, while it is repressed in response to cellular stresses including DNA damage, nutrient withdrawal, depletion of cellular energy, and hypoxia (Efeyan et al., 2012; Proud, 2004b). Moreover, it has been reported that activation of mTOR by insulin prolonged photoreceptor survival in mouse models of RP (Punzo et al., 2009). In addition, upregulation of mTOR is reported to promote retinal ganglion cell survival and axonal regeneration after optic nerve crush injury (Duan et al., 2015; Morgan-Warren et al., 2013, 2016; Morquette et al., 2015).

In this study, we show that supplementation with BCAAs enhances the utilization of glucose and the production of ATP in cultured cells, especially in high glycemic conditions. We also demonstrate significant efficacies of BCAAs against several mouse models of incurable eye diseases, such as RP and glaucoma.

## 2 Results

### 2.1 BCAA supplementation, rather than excessive glucose, raises ATP levels and protects cultured cells under stress conditions

**Fig. 1:**
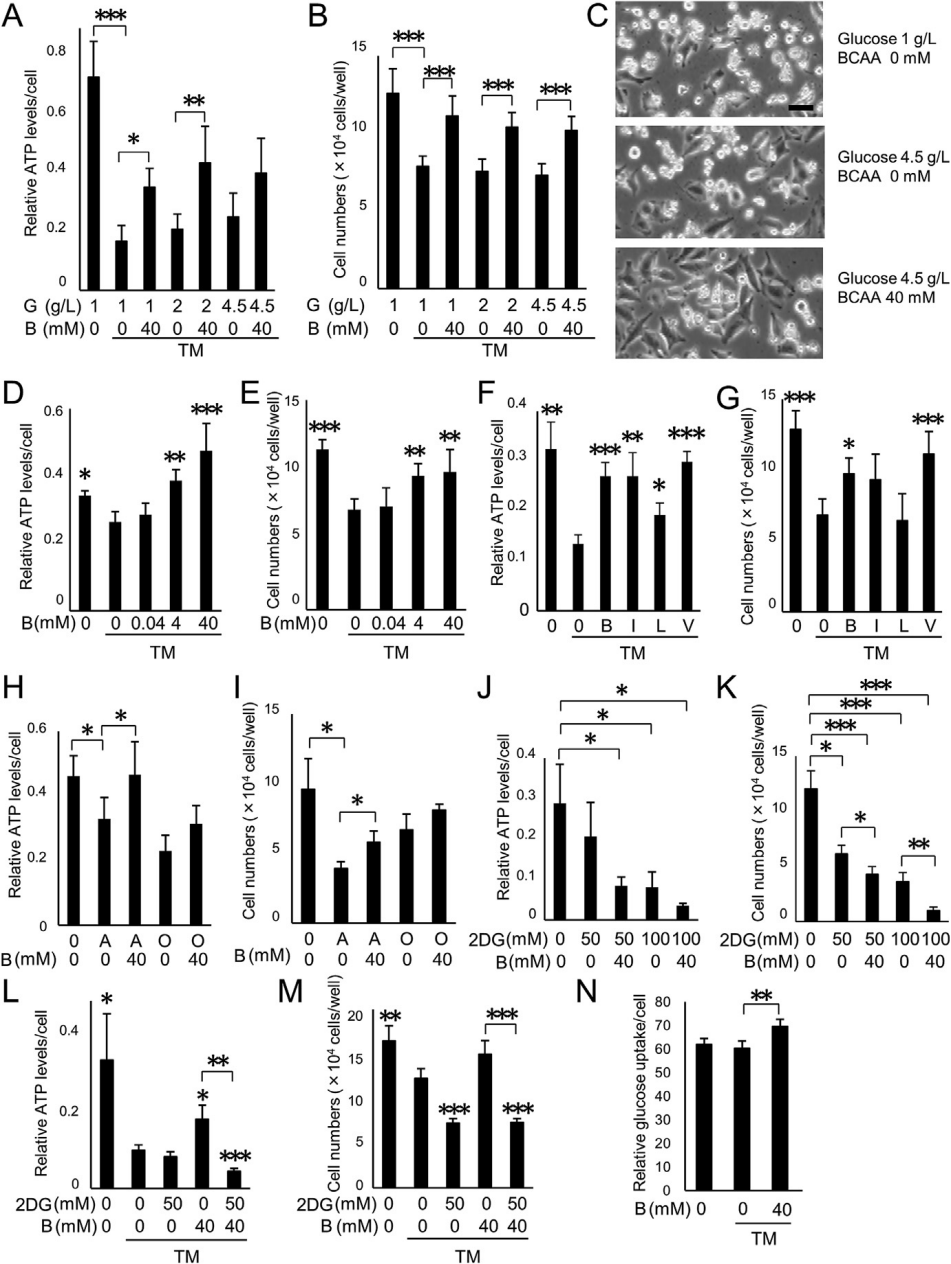
BCAAs prevent ATP decrease and cell death in cultured cells. (A–C) ATP decrease and cell death are prevented by BCAA administration but not by supplemental glucose. HeLa cells were cultured under an amino acid deficit and tunicamycin (TM) (3 μg/mL) with different concentrations of glucose (G, 1, 2 and 4.5 g/L), with or without branched chain amino acids (BCAA, B) (40 mM) for 16 hours. (A) Relative ATP levels determined by luciferase activity. *p < 0.05, **p < 0.01 and ***p < 0.001, Tukey HSD. N = 6, (B) Live cell numbers counted after trypsinization. ***p < 0.001, Tukey HSD. N = 6, (C) Representative photographs of HeLa cells. Scale bar: 20 μm. (D and E) Dose dependency of cell protective effect by BCAAs. HeLa cells were cultured with TM (3 μg/mL) and with different concentrations of BCAAs (0, 0.04, 4 or 40 mM) for 16 hours. (D) Relative ATP levels determined by luciferase activity. *p < 0.05, **p < 0.01 and ***p < 0.001, vs. TM without BCAAs, N = 6, Tukey HSD. (E) Live cell numbers counted after trypsinization. **p < 0.01 and ***p < 0.001, vs. TM without BCAAs, Tukey HSD. (F and G) Prevention of decrease of ATP and cell death by each BCAA and by the formulation of BCAAs. HeLa cells were cultured under an amino acid deficit and TM (3 μg/mL). A formulation of BCAAs, or sole isoleucine, leucine, or valine (40 mM each) were added, and cells were cultured for 16 hours. (F) Relative ATP levels determined by luciferase activity. *p < 0.05, **p < 0.01 and ***p < 0.001, vs. TM without BCAA, N = 6, Tamhane. (G) Live cell numbers counted after trypsinization. *p < 0.05 and ***p < 0.001, vs. TM without BCAA, N = 6, Tukey HSD. B, the formulation of BCAAs; I, isoleucine; L, leucine; V, valine. (H and I) Attenuation of decrease of ATP and cell death by BCAAs. HeLa cells were cultured under an amino acid deficit with antimycin (A, 30 μM) or oligomycin (O, 1μg/mL), with or without BCAAs (B, 40 mM) for 24 hours. (H) Relative ATP levels determined by luciferase activity. *p < 0.05, Tukey HSD, N = 6. (I) Live cell numbers counted after trypsinization. *p < 0.05, Tamhane. N = 6. (J and K) Effect of BCAAs on the decrease of ATP and cell death induced by 2-deoxy-D-glucose. HeLa cells were cultured with or without 2-deoxy-D-glucose (2DG, 50 or 100 mM), and with or without BCAAs (B, 40 mM) for 24 hours. (J) Relative ATP levels determined by luciferase activity. *p < 0.05, Tamhane, N = 6. (K) Live cell numbers counted after trypsinization. *p < 0.05, **p < 0.01 and ***p < 0.001, Tamhane, N = 6. (L and M) Inhibition of glycolysis abrogated the effect of BCAAs on the decrease of ATP and cell death under ER stress conditions. HeLa cells were cultured with or without 2DG (50 mM) and with or without BCAAs (B, 40 mM) in the presence of TM (3 μg/mL) for 16 hours. (L) Relative ATP levels determined by luciferase activity. *p < 0.05. **p < 0.01 and ***p < 0.001, vs. TM with neither BCAA nor 2DG, Tamhane, N = 6. (M) Live cell numbers counted after trypsinization. **p < 0.01 and ***p < 0.001, vs. TM with neither BCAAs nor 2DG, Tamhane, N = 6. (N) Calculated amount of consumed glucose/cell. HeLa cells were cultured with or without BCAAs (B, 40 mM) in the presence of TM (1 μg/mL) for 7 hours. **p < 0.01, vs. TM without BCAAs, Tukey HSD, N = 3.

A formulation of BCAAs (L-isoleucine: L-leucine: L-valine = 1: 2: 1.2), marketed as LIVACT®, has been used clinically to improve protein-nutritional status in patients with liver cirrhosis (Muto et al., 2005; Ohashi et al., 1989). We therefore used this formulation and refer to it as BCAAs hereinafter. HeLa cells were cultured under an amino acid-free condition, and then ER stress was induced by the addition of tunicamycin. As observed previously (Ikeda et al., 2014), ATP levels decreased significantly upon the addition of tunicamycin (p < 0.0001) (Fig. 1A). It is notable that the ATP levels appeared neither to recover nor increase by raising extracellular glucose concentrations (2 or 4.5 g/L, conditions similar to those after a meal or hyperglycemia; p = 0.99 and 0.72, for 2 and 4.5 g/L glucose, respectively) (Fig. 1A). Likewise, live cell numbers decreased under the ER stress condition (p < 0.0001) and were not improved by raising the glucose concentration (p = 0.997 and 0.95, for 2 and 4.5 g/L glucose, respectively) (Fig. 1B and C). On the other hand, addition of 40 mM BCAAs (see details in Materials & Methods) significantly increased the ATP levels in the presence of 1 or 2 g/L of glucose, (p < 0.0001) (Fig. 1A). Similarly, the ability of BCAAs to enhance ATP production was also observed in the presence of 4.5 g/L of glucose, although the effect did not achieve statistical significance at that concentration (because 4.5 g/L glucose alone produced a modest increase in the ATP level) (Fig. 1A). Addition of 40 mM BCAAs significantly increased the live cell numbers with all tested glucose concentrations (1, 2, and 4.5 g/L of glucose, p < 0.0001) (Fig. 1B and C). BCAAs improved the ATP levels and live cell numbers in a dose-dependent manner (ATP levels, p = 0.001 and p < 0.0001; live cell numbers, p < 0.01 and p < 0.01, for the addition of 4.0 and 40 mM BCAA, respectively, vs. controls with tunicamycin and no BCAAs) (Fig. 1D and E). These results indicate that the addition of BCAAs, but not supplementary glucose alone, enhances the production of ATP and promotes cell survival in cultured cells under ER stress.

In order to clarify which amino acid in the BCAA formulation is responsible for the ATP production, as well as the protection of cells from ER stress, HeLa cells were cultured separately with 40 mM Ile, Leu, or Val, and each culture was challenged with tunicamycin. ATP levels were strongly restored by Ile and Val and weakly by Leu (Fig. 1F). Live cell numbers increased commensurately with the degree of the recovery of ATP levels (p < 0.05 and p < 0.001, with the addition of BCAAs or Val, respectively, vs. tunicamycin challenge without BCAAs) (Fig. 1G). Among the three BCAA components, Val showed the most potent effect to promote cell survival. BCAAs also promoted cell survival similar to Val, and since BCAAs have already been used in human patients, we therefore used the LIVACT® BCAA formulation in further experiments. It is important to note that Leu, which is a potent activator of mTOR, had no beneficial effect on cell survival with this form of ER stress (Fig. 1G).

We have previously reported that inhibitors of the mitochondrial respiration chain complex are also able to induce ER stress and cell death, which were observed after the decrease in ATP levels (Nakano N. et al., 2016; Nakano M. et. al., 2017). We examined the effect of BCAAs under these conditions, and confirmed that BCAA supplementation significantly maintained ATP levels and protected cells from cell death provoked by antimycin, a specific inhibitor of mitochondrial respiratory chain complex III (p < 0.05 for antimycin treatment with the addition of BCAAs vs. antimycin treatment without BCAAs) (Fig. 1H and I). BCAAs tended, albeit not significantly, to maintain ATP levels and to protect cells from cell death upon treatment with oligomycin, a specific inhibitor of mitochondrial respiratory chain complex V (Fig. 1H and I). Interestingly, BCAAs did not lead to maintenance of ATP levels or cell protection upon inhibition of glycolysis by 2-deoxy-D-glucose (Fig. 1J and K). Furthermore, recoveries of ATP levels and cell numbers by administration of BCAAs under ER stress conditions were abrogated when glycolysis was inhibited by 2-deoxy-D-glucose (Fig. 1L and M). In addition, uptake of glucose was promoted in the BCAA-treated cells (Fig. 1N). These results indicate that addition of BCAAs enhances glycolytic ATP production, and thereby promotes cell survival in cultured cells under stress conditions.

**Fig. 2:**
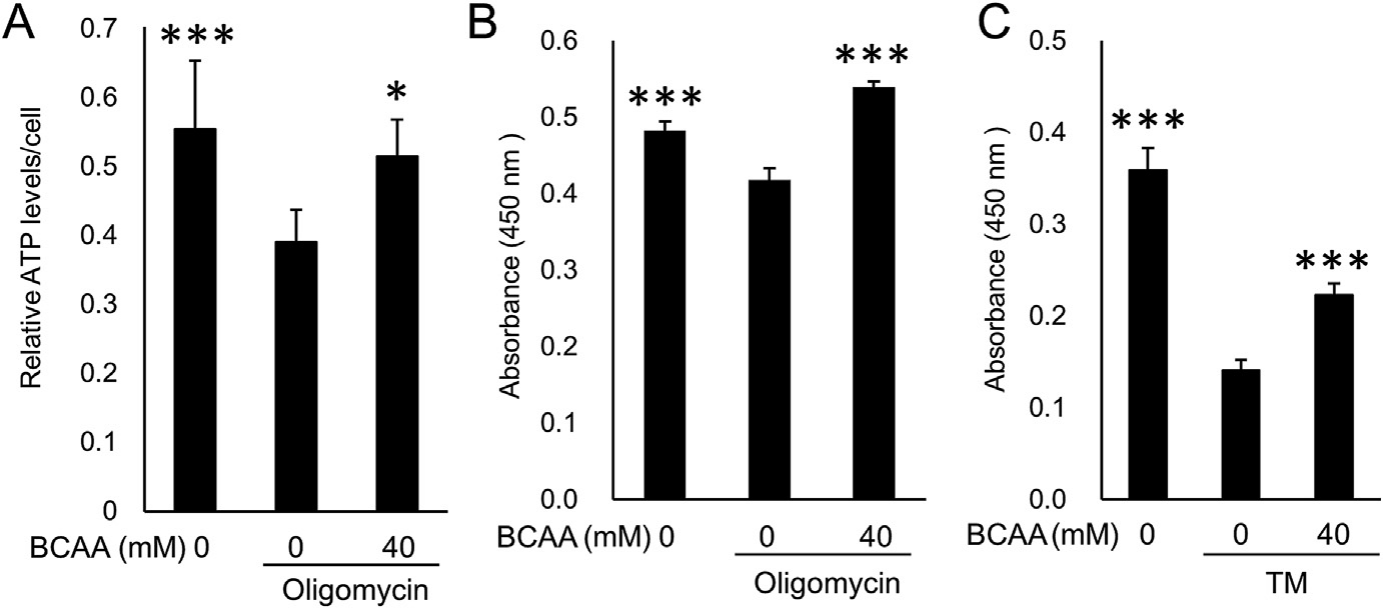
BCAAs prevent ATP decrease and cell death in photoreceptor-derived cells. (A–C) ATP decrease and cell death are prevented by BCAA administration in 661W photoreceptor-derived cells. 661W cells were cultured under an amino acid deficit and oligomycin (1 μg/mL, A and B) or tunicamycin (TM, 1 μg/mL, C) with or without BCAAs (40 mM) for 24 hours. (A) Relative ATP levels determined by luciferase activity. *p < 0.05 and ***p < 0.001, vs. oligomycin without BCAAs, Tukey HSD, N = 6. (B and C) WST (water-soluble tetrazolium salts) values reflecting relative live cell numbers are shown as optical density (OD) at 450 nm. ***p < 0.001, vs. oligomycin or TM without BCAAs, Tukey HSD, N = 3.

As HeLa cells are not an ideal model for studies of retinal degeneration, we also tested a cell line that presumably would more closely approximate cells at risk in RP. Maintenance of ATP levels and protection of cells by BCAAs was confirmed in 661W cells (Al-Ubaidi et al., 2008), a mouse photoreceptor-derived cell line (Fig. 2).

**Fig. 3:**
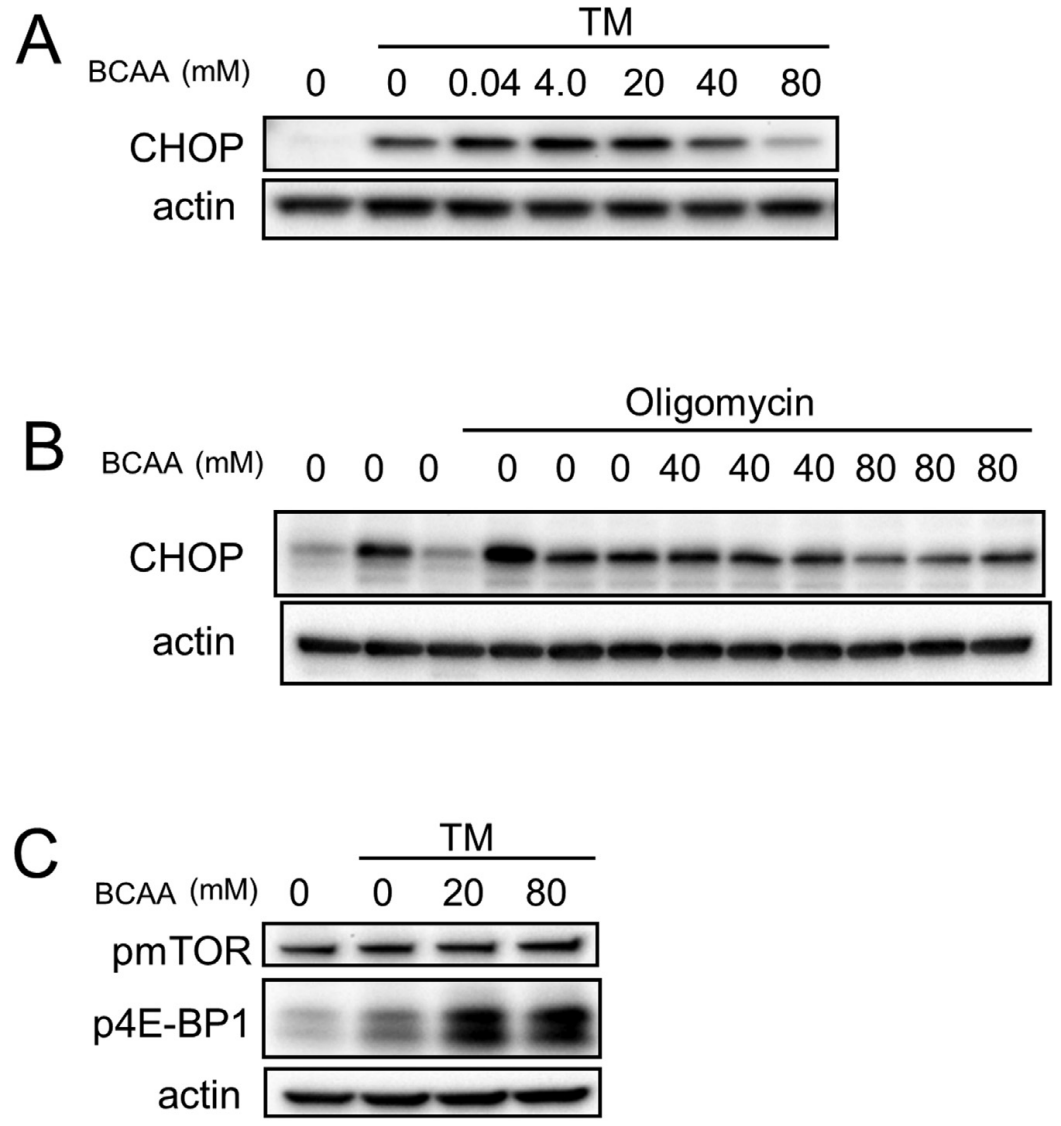
BCAAs prevent ER stress in cultured cells. (A–C) Western blot analysis of HeLa (A and C) and 661W (B) cells. (A and C) HeLa cells were cultured with TM (3 μg/mL) with or without BCAAs for 6 hours. (B) 661W cells were cultured with oligomycin (1 μg/mL) with or without BCAAs for 24 hours. (A and B) C/EBP-homologous protein (CHOP), (C) phosphorylated mammalian target of rapamycin (pmTOR, phosphorylated at S2481) and phosphorylated translational suppressor eIF4E binding protein-1 (p4E-BP1) were analyzed. Actin was used for a loading control. Complete scans of western blots are shown in Fig. S4A−C.

Next, we examined the expression of ER stress marker proteins by western blotting analyses. C/EBP-homologous protein (CHOP) (Zinszner et al., 1998) was upregulated by treatment of HeLa cells with tunicamycin, or 661W cells with oligomycin. The stress-induced elevated CHOP protein levels were significantly suppressed by the addition of high concentrations of BCAAs in HeLa cells (p < 0.01 for CHOP by the addition of 80 mM BCAAs) (Figs. 3A and S1A). Similar results were obtained in experiments using 661W cells (Figs. 3B and S1B). Together, these results suggest that treatment with BCAAs could at least partially rectify the ATP decrease and ER stress and promote cell survival *in vitro* under cell death-inducing pathological conditions.

Phosphorylated mTOR (at Ser2481), which is an activated form of mTOR, a regulator of cell survival (Hung et al., 2012; Yang and Guan, 2007), was upregulated by the addition of BCAAs (Figs. 3C and S1C). Phosphorylation of eIF4E-binding protein 1(4E-BP1), which is a target of mTOR (Hung et al., 2012; Yang and Guan, 2007), was also increased by the addition of BCAAs (Figs. 3C and S1D). Phosphorylation of another target of mTOR, phosphoprotein 70 ribosomal protein S6 kinase (p70S6K) (Hung et al., 2012; Yang and Guan, 2007), was not observed in this condition (Fig. S1E). These results suggest that activation of mTOR by BCAAs might also contribute to promote cell survival *in vivo* under cell death-inducing pathological conditions.

### 2.2 BCAA supplementation attenuates pathologies of retinal degeneration in *rd10* mice

**Fig. 4:**
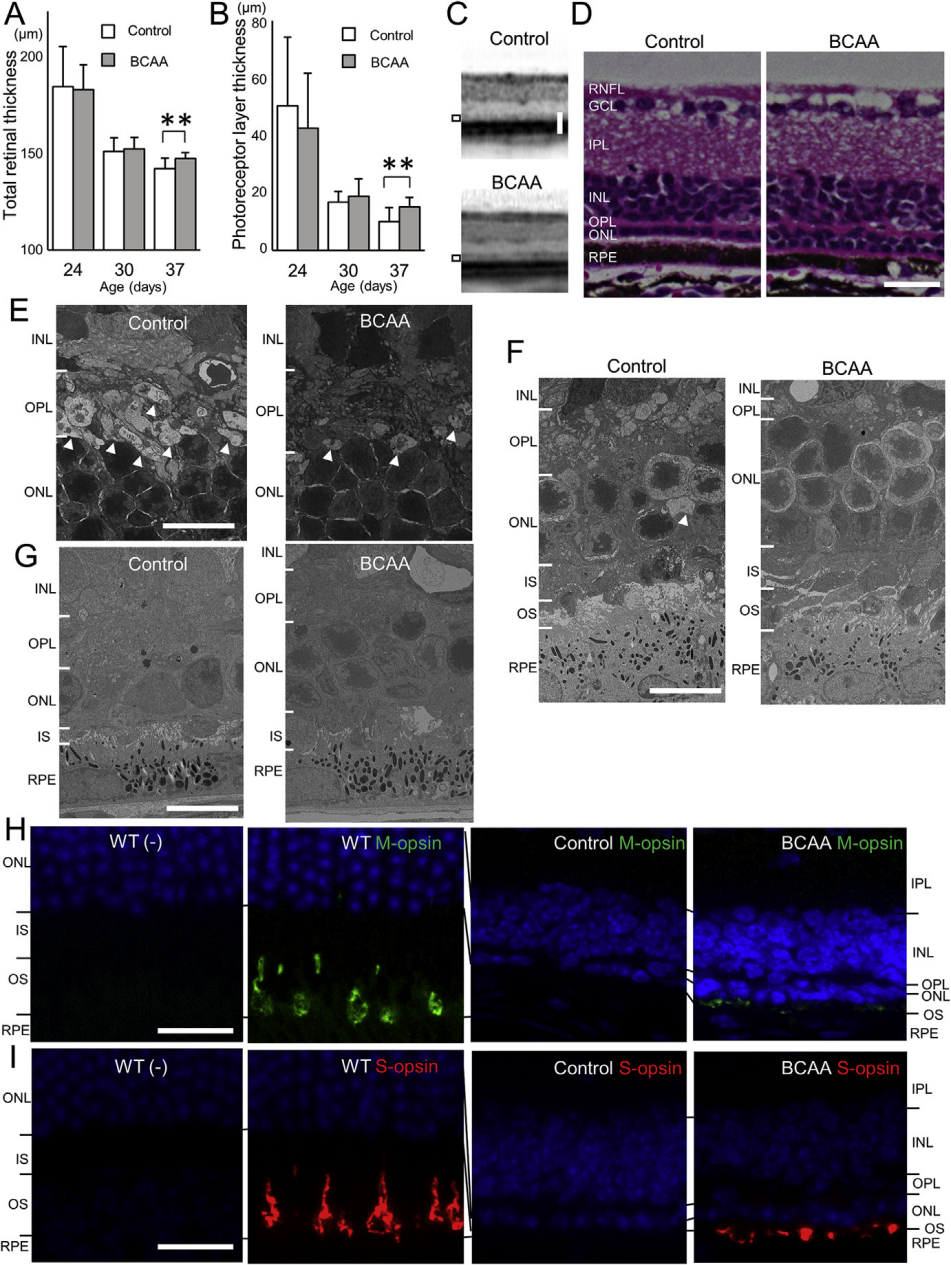
Prevention of morphological deterioration in *rd10* mice by BCAAs. (A and B) Total retinal thickness (A) and the photoreceptor layer thickness (B, the sum of the outer nuclear layer thickness and photoreceptor myoid zone, ellipsoid zone, outer segment layer thickness, indicated by black rectangles in C) in *rd10* mice measured on spectral-domain optical coherence tomography (SD-OCT) images at 24, 30, and 37 days of age. **p < 0.01, unpaired *t*-test. N = 34 eyes of 17 mice in the BCAA group and 36 eyes of 18 mice in the control group, respectively. (C) SD-OCT images of eyes in 37-day-old *rd10* mice administered supplemental BCAAs, or water as a control, which had the median photoreceptor layer thickness. (D) Vertical retinal sections of 37-day-old *rd10* mice administered BCAAs, or water as a control. (E–G) Electron microscopic images of 21-day-old (E), 30-day-old (F), and 37-day-old (G) *rd10* mice administered BCAAs, or water as a control. Intracellular vacuoles are seen in photoreceptors at the innermost ONL (white arrowheads). (H and I) Retinal sections from 37-day-old *rd10* mice administered BCAAs, or water as a control, or age-matched wild-type (WT) mice, were stained with or without an anti-M-opsin (H, green), or anti-S-opsin antibody (I, red). Nuclei were counter-stained with DAPI (blue). Abbreviations: RNFL, retinal nerve fiber layer; GCL, Ganglion cell layer; IPL, Inner plexiform layer; INL, Inner nuclear layer; OPL, Outer plexiform layer; ONL, Outer nuclear layer; IS, inner segment of the photoreceptor cell; OS, outer segment of the photoreceptor cell; and RPE, Retinal pigment epithelium. Scale bars: 50 μm in C; 20 μm in D, H and I; 10 μm in E, F and G.

**Fig. 5:**
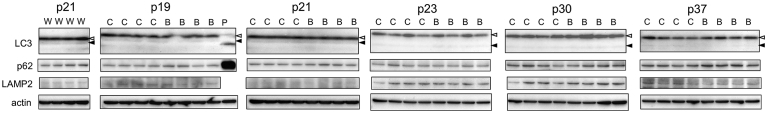
Analysis of LC3, p62, and LAMP2 showing no influences on autophagy by BCAAs in *rd10* mice. Western blot analysis of *rd10* mice treated with BCAAs, or water as a control. Neural retinas of 19-, 21-, 23-, 30-, and 37-day-old *rd10* mice were analyzed. Autophagy related protein LC3 (LC3), autophagy substrate SQSTM1 (p62) and lysosomal-associated membrane protein (LAMP) 2 were analyzed. Actin was used as a loading control. White triangle, LC3-I; black triangle, LC3-II; W, wild-type mice; C, control *rd10* mice; B, BCAA-treated *rd10* mice; P, positive control. Complete scans of western blots are shown in Fig. S4D.

We next focused on photoreceptor cell death in the retina *in vivo*. We used *rd10*, a mouse model of RP, which has a missense mutation in the *Pde6b* gene (Chang et al., 2007), to determine whether BCAAs can mitigate disease progression. Administration of BCAAs to *rd10* mice began at 7 days of age (N = 17 and 18 in BCAA-administered and control groups, respectively). Spectral domain optical coherence tomography (SD-OCT) examinations were performed to assess the total retinal thickness and the photoreceptor layer thickness, which is the sum of the outer nuclear layer (ONL), photoreceptor myoid zone, photoreceptor ellipsoid zone, and outer segment layer thickness. The total retinal thickness and the photoreceptor layer thickness did not differ significantly between groups at 24 and 30 days of age. However, both were significantly thicker in the BCAA-treated group than the non-treated control group at 37 days of age (total retinal thicknesses were 149.8 ± 3.3 and 144.2 ± 5.7 μm, respectively, p < 0.01, unpaired *t*-test, Fig. 4A; photoreceptor layer thicknesses were 15.1 ± 3.3 and 10.1 ± 4.8 μm, respectively, p < 0.01, unpaired *t*-test, Fig. 4B and C). Histological examination of retinal sections of 37-day-old *rd10* mice showed that more photoreceptor cells (represented by their nucleus numbers) remained in the ONL in the BCAA-treated mice than in the control mice (Fig. 4D). Electron microscopic analyses revealed that vacuole-like pathological structures at the innermost ONL were less numerous in BCAA-treated 21-day-old mice than the control mice (arrowheads, Fig. 4E). The numbers of vacuole-like structures were reduced in both control and BCAA-treated mice at 30 days of age, and were undetectable at 37 days of age (arrowheads, Fig. 4F and G, respectively). More photoreceptor cells remained in the BCAA-treated mice than in the control mice, even though the number was severely reduced at 37 days of age (Fig. 4G). These results are consistent with reports that demonstrated the activation of autophagy during photoreceptor cell death (Bo et al., 2015; Punzo et al., 2009), and the cell death peaking at 25 days of age in *rd10* mice (Barhoum et al., 2008). We then investigated expression levels of autophagy-related proteins, e.g. LC3, autophagy substrate SQSTM1 (p62), and lysosomal-associated membrane protein (LAMP) 2 in the retina of *rd10* mice. However, there was no significant difference in the expression of these proteins between the BCAA-treated and control *rd10* mice from 19 to 37 days of age (Fig. 5).

Immunohistochemical analyses showed that staining of M-opsin, which is a photopigment of cones sensitive to light at the middle of the visible spectrum, and staining of S-opsin, which is a photopigment of cones sensitive to short-wavelength visible light, were almost undetectable in the 37-day-old control *rd10* mice. In contrast, expression of M-opsin and S-opsin was detected in the BCAA-treated mice, albeit at much lower levels than in wild-type mice (Fig. 4H and I).

**Fig. 6:**
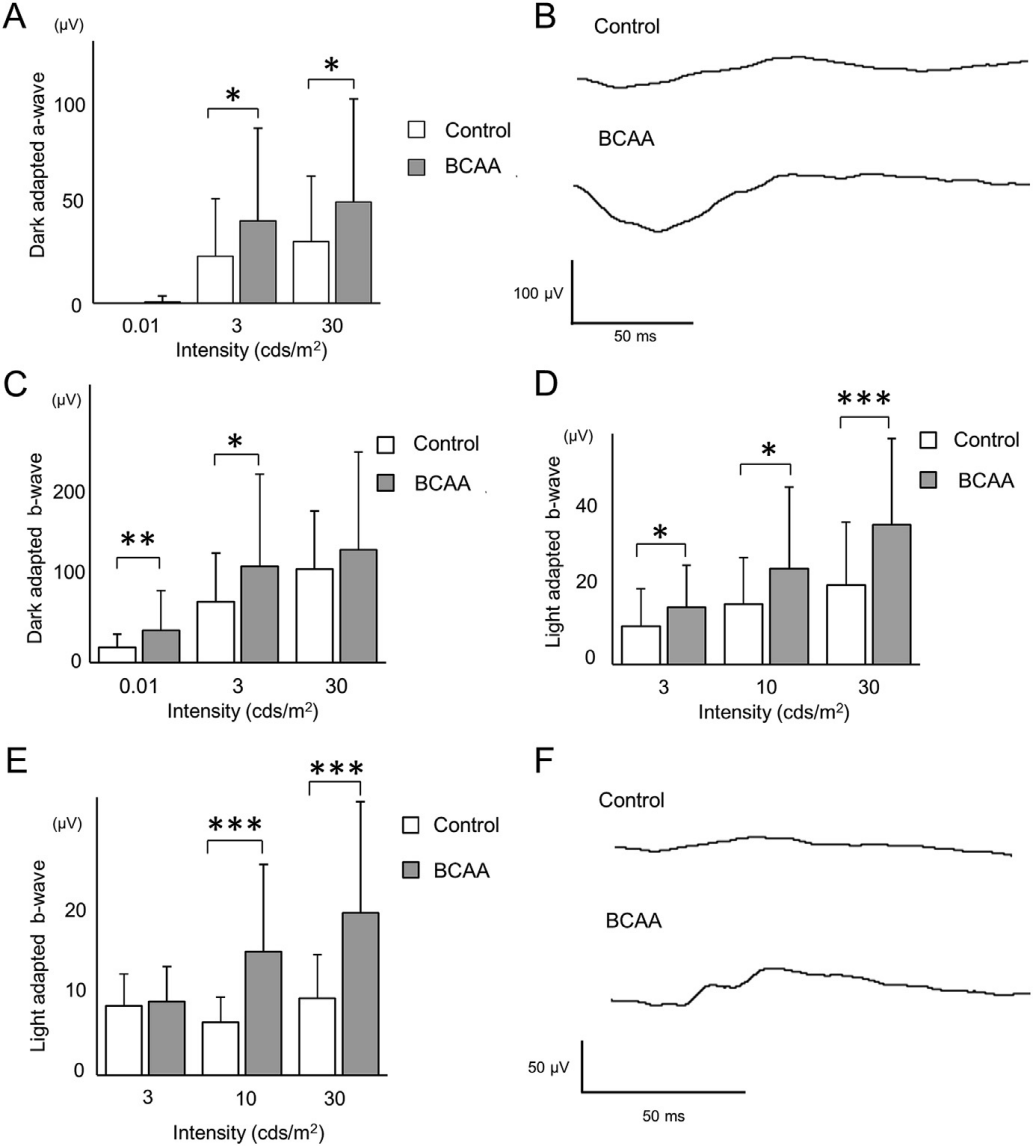
Prevention of functional deterioration in *rd10* mice by BCAAs. (A–C) Dark-adapted electroretinography (ERG) at stimulus intensities of 0.01, 3, and 30 cds/m^2^ in 24-day-old *rd10* mice administered BCAAs, or water as a control. a-wave (A) or b-wave (C) amplitudes. (B) ERG recordings at a stimulus intensity of 30 cds/m^2^ from 24-day-old *rd10* mice that showed the median b-wave amplitudes. (D–F) Light-adapted ERG at a stimulus intensities of 3, 10, and 30 cds/m^2^ in 30-day-old (D and F) or 37-day-old (E) *rd10* mice administered BCAAs or water as a control. (D and E) b-wave amplitudes in 30-day-old (D) or 37-day-old (E) *rd10* mice. (F) ERG recording at a stimulus intensity of 30 cds/m^2^ from 30-day-old *rd10* mice which showed the median b-wave amplitudes. *p < 0.05, **p < 0.01, ***p < 0.001, unpaired *t*-test.

To evaluate the effect of BCAAs on photoreceptor functions, scotopic electroretinography, which measures retinal function in the dark, was performed at 24 days of age, and photopic electroretinography, which measures retinal function in the light, was performed at 30 and 37 days of age. At 24 days of age, the scotopic electroretinography a-wave, which reflects photoreceptor functions (Hood and Birch, 1990a, 1990b), was significantly larger in the BCAA-treated group than the control group (43.2 ± 48.6 and 24.7 ± 3.2 μV, respectively, p = 0.03, unpaired *t*-test, at stimulus intensity of 3 cds/m^2^; and 53.0 ± 54.1 and 32.3 ± 34.4 μV, respectively, p = 0.03, unpaired *t*-test, at stimulus intensity of 30 cds/m^2^, Fig. 6A and B). The scotopic electroretinography b-wave at a stimulus intensity of 0.01 cds/m^2^, which reflects rod-system response (McCulloch et al., 2015), was significantly larger in the BCAA-treated group than the control group (40.1 ± 50.2 and 18.8 ± 16.7 μV, respectively, p < 0.01, unpaired *t*-test, Fig. 6C). The b-wave at a stimulus intensity of 3 cds/m^2^, which is derived from bipolar cells (Hood and Birch, 1996), was also significantly larger in the BCAA-treated group than the control group (121.3 ± 115.9 and 76.4 ± 61.3 μV, respectively, p = 0.01, unpaired *t*-test, Fig. 6C). At 30 and 37 days of age, the b-wave of photopic electroretinography was significantly larger in the BCAA-treated group than the control group (14.8 ± 11.0 and 10.0 ± 9.7 μV, respectively, p = 0.03, unpaired *t*-test, at stimulus intensity of 3 cds/m^2^; 24.9 ± 21.2 and 15.7 ± 12.0 μV, respectively, p = 0.01, unpaired *t*-test, at stimulus intensity of 10 cds/m^2^; and 36.3 ± 22.4 and 20.6 ± 16.3 μV, respectively, p < 0.001, unpaired *t*-test, at stimulus intensity of 30 cds/m^2^, at 30 days of age, Fig. 6D, E and F). These experiments show that BCAA treatment protected photoreceptors with regard to both morphology and function in *rd10* mice.

### 2.3 Treatment of adult *rd12* mice with BCAAs attenuates disease features at a later disease stage

**Fig. 7:**
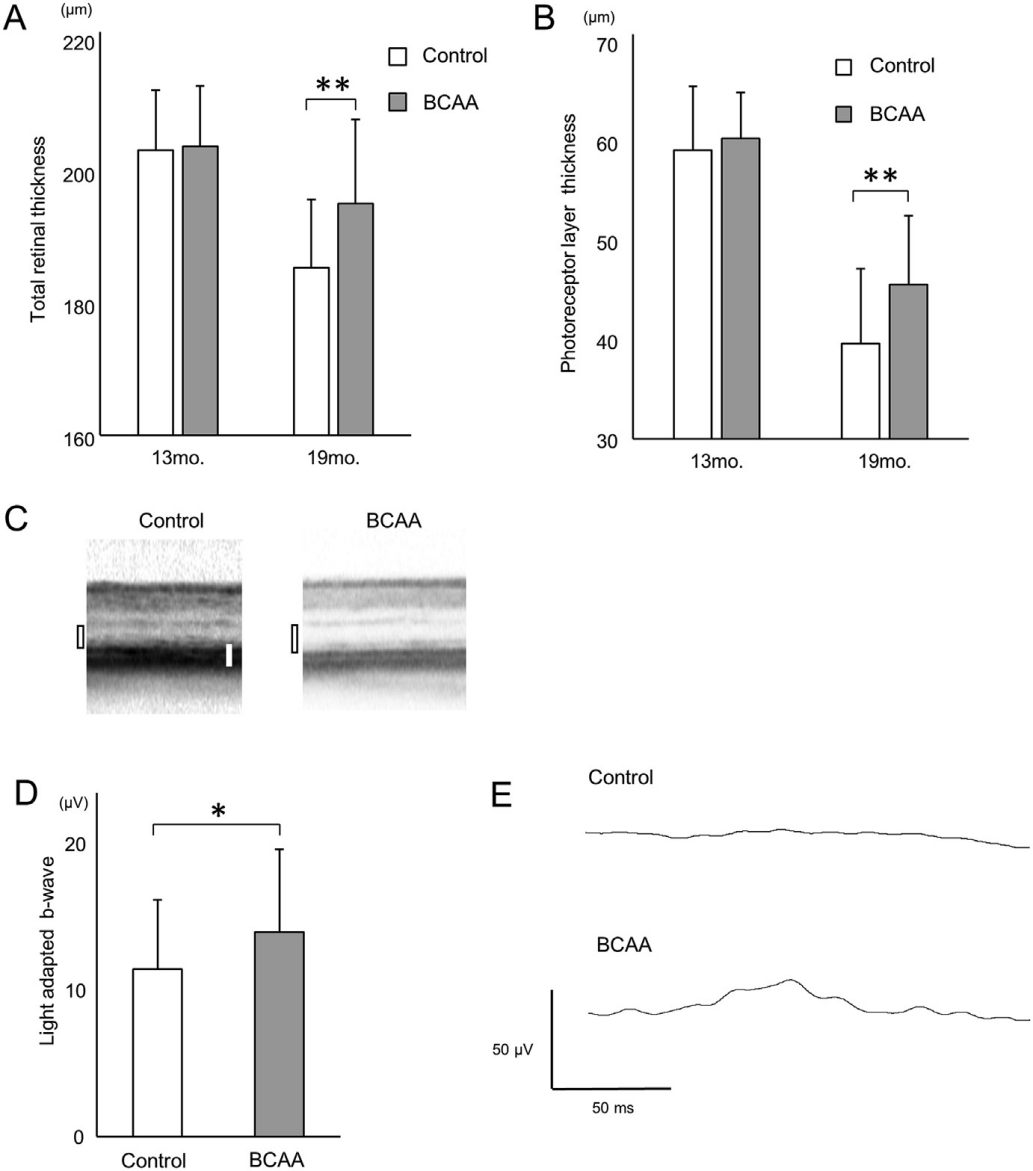
Prevention of morphological and functional deterioration in later disease stage *rd12* mice by BCAAs. (A and B) Total retinal thickness (A) and the photoreceptor layer thickness (B, the sum of outer nuclear layer thickness and photoreceptor myoid zone, ellipsoid zone, outer segment layer thickness, black rectangles in C) of *rd12* mice administered BCAAs or water as a control, measured on SD-OCT images. (C) SD-OCT images of 19-month-old *rd12* mice retinas. Scale bar (white bar): 50 μm. (D) b-wave amplitudes of light-adapted ERG at a stimulus intensity of 10 cds/m^2^ in 19-month-old *rd12* mice administered BCAAs or water as a control. (E) Light-adapted ERGs at a stimulus intensity of 10 cds/m^2^ in 19-month-old *rd12* mice. *p < 0.05, **p < 0.01, unpaired *t*-test.

In order to ascertain whether BCAAs can protect photoreceptors at a later stage of retinal degeneration, we used *rd12*, another mouse model for retinal degeneration, which has a nonsense mutation in the *Rpe65* gene (Pang et al., 2005). Thirteen-month-old *rd12* mice, when retinal degeneration had already started (Hasegawa et al., 2016a), were assigned to two groups, the BCAA-treated and non-treated control groups (N = 17 mice for each). The BCAA-treated group was allowed *ad libitum* access to water containing BCAAs, whereas the control group was allowed *ad libitum* access to water without BCAAs. At 13 months of age, total retinal thickness and photoreceptor layer thickness, which were measured on SD-OCT images, were not significantly different (p = 0.40 and p = 0.20, respectively, unpaired *t*-test). After 6 months of administration of BCAAs (at 19 months of age), total retinal and photoreceptor layers were significantly thicker in the BCAA-treated group than the non-treated control group (total retinal thickness, 194.4 ± 12.6 and 184.9 ± 10.2 μm, respectively, p < 0.01, unpaired *t*-test; photoreceptor layer thickness, 45.3 ± 6.8 and 39.4 ± 7.4 μm, respectively, p < 0.01, unpaired *t*-test, Fig. 7A, B and C).

In order to assess intact retinal function, photopic electroretinography was recorded at the age of 19 months. b-wave amplitudes of photopic electroretinography were very small in both groups, but they were significantly larger in the BCAA-treated group than the non-treated control group (14.9 ± 6.0 and 12.2 ± 5.0 μV, respectively, p = 0.046, at a stimulus intensity of 10 cds/m^2^, Fig. 7D and E).

These results indicate that administration of BCAAs, even started after the onset of the disease, provides a modest protection of photoreceptor cells both morphologically and functionally in *rd12*, a mouse model of retinal degeneration.

### 2.4 Administration of BCAAs to glaucoma model mice attenuates retinal ganglion cell death

**Fig. 8:**
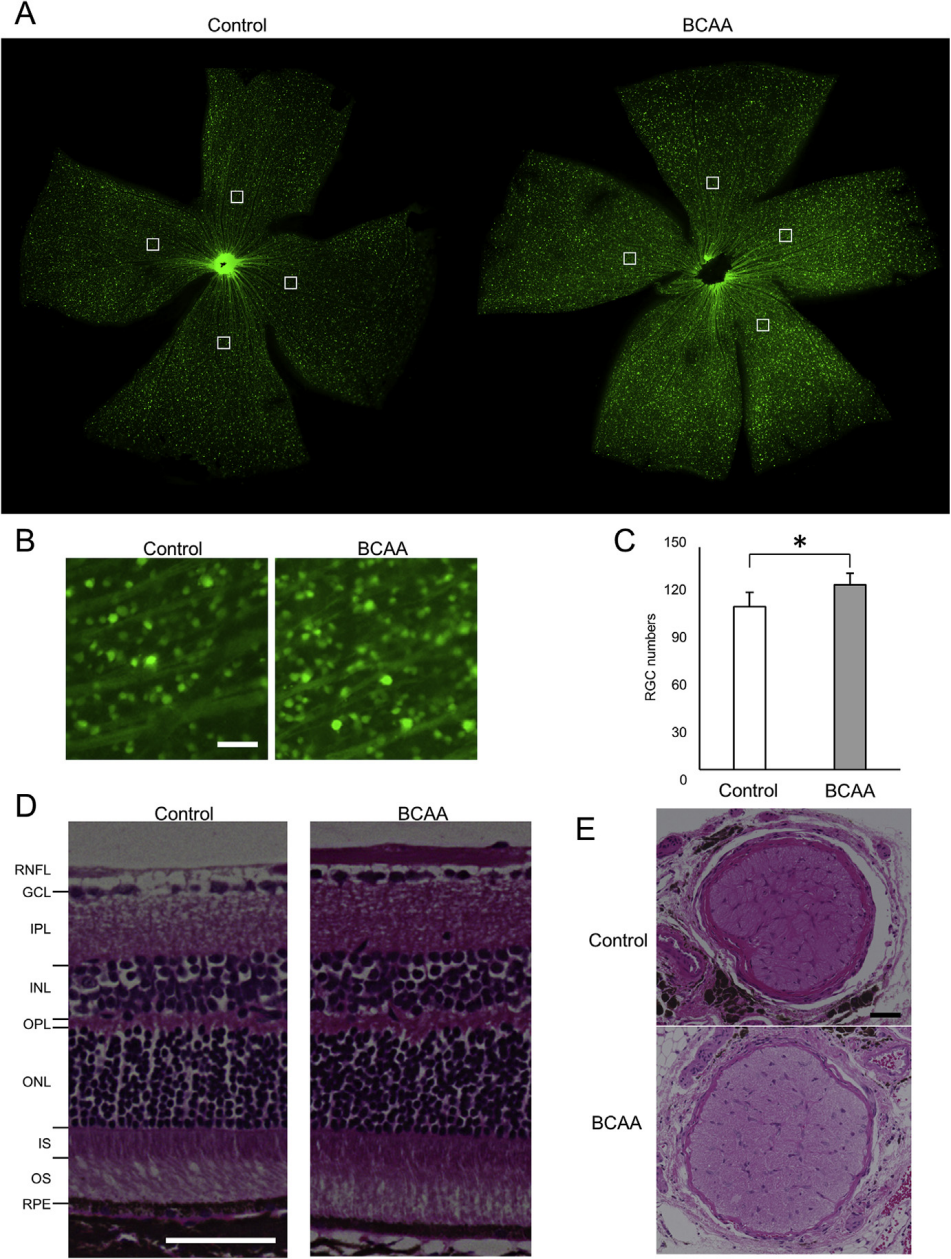
Prevention of retinal ganglion cell death in GLAST (+/−) mice by BCAAs. (A–C) Retinal flat mount of 12-month-old GLAST (+/−):Thy1-CFP mice administered BCAAs, or water as a control. (A) The CFP-positive retinal ganglion cells were counted at a distance of 1200 μm from the optic nerve head center. The white squares (250 μm squares) indicate areas in which CFP-positive retinal ganglion cells were counted. (B) Magnified images of retinal flat mounts from GLAST (+/−):Thy1-CFP mice that showed the median retinal ganglion cell counts. (C) Numbers of CFP-positive retinal ganglion cells on the retinal flat mount. N = 6 eyes of 6 mice in the BCAA group and N = 4 eyes of 4 mice in the control group, respectively, *p < 0.05, unpaired *t*-test. (D) Hematoxylin and eosin (HE)-stained vertical retinal sections of 12-month-old GLAST (+/−) mice administered BCAAs, or water as control. Abbreviations: RNFL, retinal nerve fiber layer; GCL, ganglion cell layer; IPL, Inner plexiform layer; INL, Inner nuclear layer; OPL, Outer plexiform layer; ONL, Outer nuclear layer; IS, inner segment of the photoreceptor cell; OS, outer segment of the photoreceptor cell; and RPE, Retinal pigment epithelium. (E) HE-stained optic nerve cross-sections from 18-month-old GLAST (+/−) mice that showed the median cross-section areas. Scale bars: 50 μm in B, D and E.

We then examined if administration of BCAAs can prevent or delay retinal ganglion cell death *in vivo.* Glutamate-aspartate transporter (GLAST) knockout mice manifest chronic retinal ganglion cell loss and are used as a glaucoma model (Harada et al., 2007). We created GLAST (+/−) mice expressing cyan fluorescent protein (CFP) in the retinal ganglion cells (referred to as GLAST (+/−)-CFP mice) (Feng et al., 2000). Two groups of GLAST (+/−)-CFP mice were fed normal diets, and BCAAs were daily administered to the BCAA-treated group (see Materials & Methods). At 12 months of age, retinal flat mounts showed that CFP-positive retinal ganglion cells were significantly better retained in the BCAA-treated group than the control group without BCAAs (124.8 ± 7.5 and 110.0 ± 9.6 cells, respectively, p = 0.016, unpaired *t*-test, Fig. 8A, B and C). Hematoxylin and eosin (HE)-stained retinal sections from BCAA-treated mice had a thicker retinal nerve fiber layer than sections from the control group (Fig. 8D). The cross-sectional area of the optic nerve, which is presented as the median value, was larger in the BCAA-treated group than the control group (70,673 μm^2^ (N = 4) and 45,352 μm^2^ (N = 5), respectively) (Fig. 8E). These data demonstrate that BCAAs protected retinal ganglion cells in the glaucoma model.

### 2.5 BCAA administration reduces ER stress, activates mTOR, and suppresses apoptosis in RP and glaucoma mouse models

**Fig. 9:**
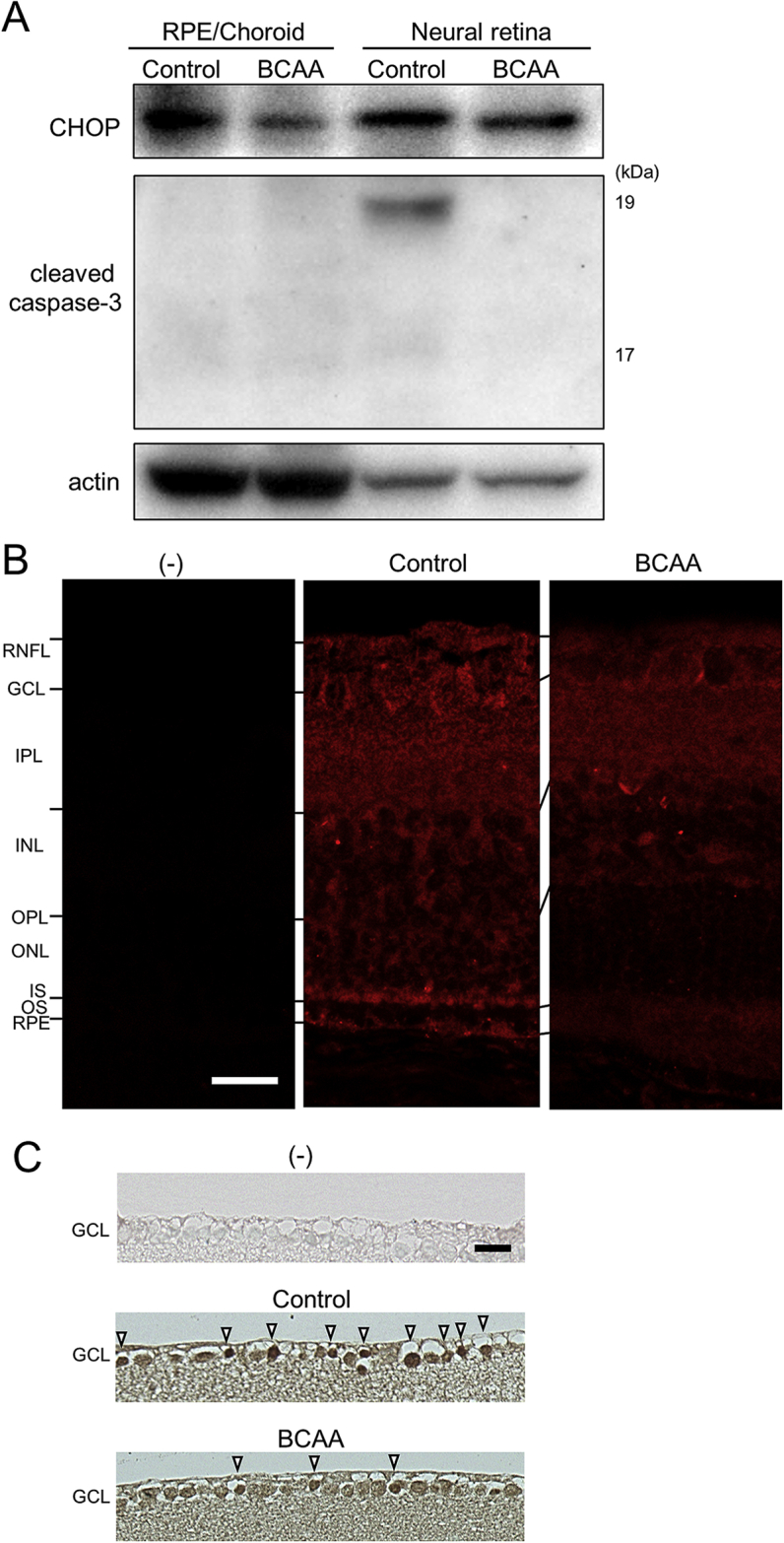
Suppression of ER stress by BCAAs leads to suppression of cleaved caspase-3 in mouse models. (A) Western blot analysis of 19-month-old *rd12* mice treated with BCAAs, or water as a control. Extracts from dissected neural retinas and the combination of retinal pigment epithelium (RPE), choroid, and sclera (RPE/choroid) were analyzed with an anti-CHOP or cleaved caspase-3 antibody. Complete scans of western blots are shown in Fig. S4E. (B) Vertical retinal sections of 21-day-old *rd10* mice stained with or without an anti-CHOP antibody (red). Abbreviations: RNFL, retinal nerve fiber layer; GCL, Ganglion cell layer; IPL, Inner plexiform layer; INL, Inner nuclear layer; OPL, Outer plexiform layer; ONL, Outer nuclear layer; IS, inner segment of the photoreceptor cell; OS, outer segment of the photoreceptor cell; and RPE, Retinal pigment epithelium. (C) Vertical retinal sections of 18-month-old GLAST (+/−) mice stained with or without an anti-CHOP antibody. Note that more cells remained in the GCL of BCAA-treated mouse than in that of control mouse. Note also that more cells were strongly stained by the CHOP antibody (arrowheads) in control mouse than in BCAA-treated mouse. Scale bars: 20 μm in B and C.

We investigated potential mechanisms of retinal protection by the administration of BCAAs in these animal models of retinal diseases. Western blot analyses of retinal samples from 19-month-old *rd12* mice revealed that the expression of CHOP (Zinszner et al., 1998) was reduced both in neural retinas and the mixed samples of retinal pigment epithelium and choroid (RPE/choroid) in the BCAA-treated mice, compared to the non-treated control (Figs. 9A, S2A-D). Immunohistochemical analysis of retinas from 21-day-old *rd10* mice showed that CHOP expression was reduced at the outer layers of the retina in the BCAA-treated group, compared to those in the non-treated control group (the outer nuclear layer, inner segment of the photoreceptor and retinal pigment epithelium, Fig. 9B). In BCAA-treated 18-month-old GLAST (+/−) mice, CHOP expression was also reduced in the retinal ganglion cells, compared to the non-treated control mice (Fig. 9C).

Expression of phosphorylated-mTOR (pmTOR) in the RPE/choroid and in the neural retina showed a tendency to be suppressed in control 19-month-old *rd12* mice, relative to wild-type mice, and showed a tendency to be partially restored in BCAA-treated *rd12* mice (Fig. S2E). p70S6K (Hung et al., 2012; Yang and Guan, 2007), one of the major targets of mTOR, showed a tendency to be more activated in the neural retinas of BCAA-treated *rd12* mice than controls (Fig. S2F). To ascertain whether suppression of ER stress by BCAA administration may forestall apoptosis, cleaved caspase-3, which is generated in apoptotic cells, was analyzed. The protein levels of cleaved caspase-3 were lower in the neural retinas of BCAA-treated *rd12* mice than the non-treated controls (Fig. 9A). These results are consistent with the idea that BCAAs suppress ER stress, which in turn results in reduction of apoptosis in these animal models of retinal diseases.

## 3 Discussion

In this study, we showed that BCAAs worked against cell death *in vitro* and *in vivo*. In cultured cells, BCAAs enhanced the utilization of glucose to produce ATP and suppressed cell death under ER stress-induced conditions. In glaucoma and retinal degeneration, ER stress has been proposed as an underlying mechanism. Consistently, administration of BCAAs to mouse models of RP and glaucoma suppressed cell death of retinal neuronal cells, including the retinal ganglion cells and photoreceptors, and preserved visual functions to a certain extent.

We have shown that maintenance of the ATP level is an effective therapeutic strategy in preventing cell death in mouse models of RP and glaucoma. In our previous studies, we reported the development of KUSs as novel inhibitors of the ATPase activities of VCP, the most abundant soluble ATPase in all types of cells (Ikeda et al., 2014). Indeed, KUSs manifested significant efficacies in mouse models of RP (Hasegawa et al., 2016b; Ikeda et al., 2014) and glaucoma (Nakano et al., 2016). We subsequently looked for compounds that would enhance ATP production (Nakano et al., 2017). Ideally, such materials should have no toxicity in humans. From this point of view, various nutritional supplements, which are daily taken by humans, would be good candidates. Carbohydrates (sugars), lipids, and proteins (amino acids) are three major nutrients, or energy sources. Among them, sugars and lipids would not be suitable, because the intake of high amounts of sugars and lipids raises the risk of metabolic syndrome and obesity. In contrast, high intake of amino acids does not appear to be harmful to humans. For example, supplemental BCAAs have been taken daily by athletes to improve their condition. In addition, BCAAs, as LIVACT®, has been used as a therapeutic drug for patients with liver cirrhosis. In the literature, BCAAs have been shown to improve glucose metabolism in cirrhotic rat liver and improve glucose uptake in rat skeletal muscle (Doi et al., 2005; Matsumura et al., 2005; Nishitani et al., 2002). We then decided to examine the efficacies of BCAAs to enhance ATP production.

As expected, BCAA supplementation maintained ATP levels in cells treated with tunicamycin, an ER stress inducer, which in turn reduced ER stress levels and protected cells from cell death. We originally assumed that BCAAs were metabolized as sources of ATP production. However, BCAAs functioned to enhance glucose uptake and utilization, because the effects were canceled by the addition of 2-deoxy-D-glucose, an inhibitor of glycolysis. It was surprising to observe that glucose supplementation itself did not suppress the decrease in ATP levels; nor did it inhibit cell death under the same stress condition where BCAA supplementation did (Fig. 1). BCAAs are known to activate mTOR. In our ER stress-inducing cell culture model, however, administration of Leu alone, which is a potent activator of mTOR, could not apparently inhibit cell death. Nevertheless, we found that BCAAs activated mTOR in certain levels in the retina of the mouse disease models (Fig. S2E and F). Therefore, it remains possible that activation of mTOR by BCAAs would also contribute to the delay of retinal neuronal cell death *in vivo*.

We confirmed that peritoneal BCAA administration increased the BCAA concentration in eyes in a dose-dependent manner (Fig. S3). In the pilot experiment with *rd10* mice given two doses (1.5 g/kg/day or 4.5 g/kg/day) of BCAAs, we found that the 1.5 g/kg/day BCAA administration appeared to be sufficient to achieve neuroprotective effects; administration of 1.5 g/kg/day BCAAs to rodents has been reported to maintain almost the same plasma concentration of BCAAs as patients given 12 g of LIVACT® per day, which is the clinically accepted dose for patients with liver cirrhosis (Matsumura et al., 2005).

It is notable that BCAAs worked against the progression of retinal degeneration, even when treatment started at later stages of the diseases, when photoreceptor degeneration had progressed considerably (Fig. 7), and that BCAAs suppressed degeneration of cone photoreceptors, which are important in central vision and in quality of vision (Fig. 4). These data suggest that administration of BCAAs, as the formulation of LIVACT®, at the clinically used dosage would be effective to delay disease progression in patients with RP or glaucoma. We found that among the constituents of BCAAs, Ile and Val were better in the suppression of ATP loss than Leu (Fig. 1). This is consistent with a report that Ile has a stronger glucose uptake stimulating effect than Leu (Doi et al., 2005). Another report demonstrated that supplemental administration of Leu caused food intake reduction and growth suppressing effects, which were reversed by supplementation with Ile and Val (Harper et al., 1984). Thus, BCAAs as LIVACT®, containing Leu, Ile, and Val, have been used in patients with liver cirrhosis (Muto et al., 2005) and are considered to possess very high levels of safety (Holecek, 2013; Muto et al., 2005).

In conclusion, BCAAs worked against cell death via enhancing ATP production, supporting our proposal that ATP maintenance is an effective therapeutic strategy for protecting cells from cell death-inducing insults. BCAAs protected not only rod photoreceptors but also cone photoreceptors and worked against cell death even when the administration was started at later stages of retinal degeneration in a mouse model of retinal degeneration. BCAA administration also suppressed the loss of retinal ganglion cells in a glaucoma mouse model. BCAAs show great potential for use as an easily available and effective therapeutic strategy for incurable eye diseases, including RP as well as glaucoma.

## 4 Materials & methods

### 4.1 Cell culture

HeLa cells were cultured in Dulbecco's modified Eagle's medium (DMEM) containing 4.5 g/L of glucose without amino acids (048-333575; Wako Pure Chemical Industries, Ltd.) without serum, or in DMEM without glucose without amino acids (specially ordered, Funakoshi Co., Ltd.). Glucose was added (0, 1, 2 or 4.5 g/L) when the cells were cultured in DMEM without glucose without amino acids. BCAAs (L-isoleucine: L-leucine: L-valine = 1:2:1.2, the same as LIVACT®; Ajinomoto Co.) were added to the medium (0, 0.04, 4.0, 20, 40, or 80 mM, calculated as 126.829 g/mol as the molecular weight, which was calculated from the respective molecular weights and the abundance ratio in the formulation). In experiments with separate additions, Ile, Leu, or Val was added at a concentration of 40 mM. Tunicamycin (Nacalai Tesque, 3 μg/mL) was added to induce ER stress. Antimycin (Sigma, 30 μM) or oligomycin (Sigma, 1 μg/mL) was added to inhibit mitochondrial respiratory chain complex III or complex V. 2-deoxy-D-glucose (Nacalai Tesque, 50, or 100 mM) was added as an inhibitor of glycolysis. Cells were cultured for 16 hours in the medium with or without tunicamycin, and with or without BCAAs for measurements of relative ATP levels or live cell numbers. Cells were cultured for 24 hours in the medium with or without antimycin, oligomycin, or 2-deoxy-D-glucose, and with or without BCAAs for measurements of relative ATP levels or live cell numbers. Relative ATP levels in cultured cells were measured by luciferase activities with an ARVO multilabel counter using ATP assay reagent for cells (Toyo B-net) (Ikeda et al., 2014). Live cell numbers were measured with a TC10 automated cell counter (Bio RAD) after trypsinization. Cells were cultured for 6 hours in the medium with or without tunicamycin and with or without BCAAs for western blotting analysis. Glucose concentration was measured using a Glucose Colorimetric Assay Kit (BioVision), and consumption of glucose/cell was calculated.

661W cells were kindly provided by Dr. Muayyad R. Al-Ubaidi (University of Houston, Houston, TX, USA). Cells were cultured for 24 hours in the medium with or without oligomycin (1 μg/mL) or tunicamycin (1μg/mL), and with or without BCAAs for measurements of relative ATP levels or viability of the cells using WST (water soluble tetrazolium salts)-8 reagent (Cell count reagent SF, Nacalai Tesque, Kyoto, Japan).

### 4.2 Experimental animals

This study was conducted in accordance with the Association for Research in Vision and Ophthalmology (ARVO) Statement for the Use of Animals in Ophthalmic and Vision Research. All protocols were approved by the Institutional Review Board of Kyoto University Graduate School of Medicine (MedKyo 14213, 15531, 16501, 17272). Model mice for retinal degeneration, *rd10* mice (Chang et al., 2007) and *rd12* mice (Pang et al., 2005) and B6. Cg-Tg (Thy1-CFP) 23Jrs/J mice, in which retinal ganglion cells express CFP (Feng et al., 2000; Murata et al., 2008), were obtained from the Jackson Laboratory. Glutamate-aspartate transporter (GLAST) knockout mice, which manifest retinal ganglion cell loss and are used as a glaucoma model (Harada et al., 2007), were a gift from Dr. Koichi Tanaka (Tokyo Medical and Dental University). GLAST knockout mice and Thy1-CFP mice were crossed to obtain GLAST (+/−):Thy1-CFP mice (Nakano et al., 2016). Mice were maintained in a 14-hour light/10-hour dark cycle and were fed *ad libitum*. Before SD-OCT imaging or electroretinography acquisition, mice were anesthetized with an intraperitoneal injection of a ketamine (70 mg/kg)/xylazine (14 mg/kg) mixture. Pupils were dilated with tropicamide and phenylephrine eye drops (0.5% each).

### 4.3 Administration of BCAAs

We confirmed that BCAA administration increased the BCAA concentration in eyes in a dose-dependent manner (Fig. S3). One-month-old GLAST (+/−):Thy1-CFP mice were assigned to either a BCAA group or a control group. BCAA group mice had intraperitoneal administration of BCAAs (0.375 g/kg/day) from 1 month to 2 months of age and then started oral medication with BCAAs from 2 months of age. These mice had *ad libitum* access to water containing 7.5 g/L of BCAAs. For oral *ad libitum* administration of BCAAs, we measured the amount of water decrement in the water bottles and weighed the mice to calculate the administration dosage. The administration with *ad libitum* access to water containing 7.5 g/L was about 1.5 g/kg/day with an assumption that the mice drank all the amount of the decrement. Control group mice had intraperitoneal administration of saline from 1 month to 2 months of age and were provided with access to water *ad libitum* after weaning. Seven-day-old *rd10* mice were assigned to either the BCAA group or control group and had daily intraperitoneal administration of BCAAs (0.75 g/kg, twice a day: 1.5 g/kg/day) or saline, and the BCAA group mice were then switched to oral medication with *ad libitum* access to the water containing BCAAs at 24 days of age. Other 7-day-old *rd10* mice had daily intraperitoneal administration of BCAAs (0.75 g/kg, twice a day: 1.5 g/kg/day) and then were assigned to either the BCAA group or an increased dose BCAA group at weaning. These mice had *ad libitum* access to water containing 7.5 g/L of BCAAs (BCAA group, about 1.5 g/kg/day) or 22.5 g/L of BCAAs (increased dose BCAA group, about 4.5 g/kg/day) to examine dose-dependency of the effect of BCAAs. We confirmed that the increased dose administration of BCAAs was not superior to the 1.5 g/kg/day of BCAA administration. Based on these results, we considered that the 1.5 g/kg/day dose is sufficient to show neuroprotective efficacy *in vivo* and used that dosage for *in vivo* experiments thereafter. Thirteen-month-old *rd12* mice were assigned to either a BCAA group or a control group and the BCAA group started oral medication with *ad libitum* access to water containing 7.5 g/L of BCAAs (BCAA group, estimated intake was thus about 1.5 g/kg/day) while the control group had *ad libitum* access to water.

### 4.4 SD-OCT acquisition

Speckle noise-reduced SD-OCT with the eye-tracking function based on Spectralis® HRA+OCT (*Multiline* OCT; Heidelberg Engineering) (Nakano et al., 2011) was used to obtain fundus images. A 25-diopter adaptor lens was placed on the objective lens of the *Multiline* OCT in order to focus onto the mouse retina. Vertical B-scans through the optic nerve head were obtained by averaging one hundred individual B-scans (manufacturer-set maximum), and 19 vertical B-scans evenly spaced over a 30° × 15° area through the optic nerve head were obtained by averaging 50 individual B-scans for volume mapping.

### 4.5 Analysis of SD-OCT images

In *rd10* and *rd12* model mice, we assessed total retinal thickness, which was evaluated as the distance between the inner limiting membrane and the outer side of the Bruch's membrane, and photoreceptor layer thickness, which was evaluated as the sum of ONL thickness and photoreceptor myoid zone, photoreceptor ellipsoid zone, and outer segment layer thickness (black rectangles in Figs. 4C and 7C). The vitreoretinal interface, outer plexiform layer/ONL, RPE anterior border, and Bruch's membrane posterior border were manually determined and then built-in software of the Spectralis HRA-OCT was used to measure the thickness of each layer. A volume map around the optic nerve head was used to assess total retinal thickness within 366 μm of the optic nerve head in all directions (circular area) and the circular area within 122 μm of the optic nerve head was excluded from analyses. The values measured at the upper, lower, right, and left regions were averaged. Photoreceptor layer thickness was measured at 244 μm above and below the center of the optic nerve head using a single vertical scan, and then these two measurements were averaged to obtain the final photoreceptor layer thickness. Eyes were excluded from the retinal thickness measurements when retinal detachment was observed.

### 4.6 Electroretinography recording and analysis

A gold-loop corneal electrode with a light-emitting diode (Mayo Corp.) was used to record electroretinography. A reference electrode was placed in the mouth, and a ground electrode was placed in the anus. A light-emitting diode stimulator (Mayo Corp.) was used to produce stimuli. Scotopic electroretinography was recorded after overnight dark adaptation with stimulus intensity of 0.01 (rod response), 3 (mixed cone and rod response), and 30 cds/m^2^ (McCulloch et al., 2015). Photopic electroretinography was recorded with stimulus intensity of 3, 10, and 30 cds/m^2^ and a background illumination of 30 cd/m^2^ (McCulloch et al., 2015). The electroretinography response was amplified (PowerLab 2/25; AD instruments), and up to 4 responses were averaged in scotopic electroretinography, and 30 to 50 responses were averaged in photopic electroretinography, to obtain the final electroretinography waveform. The stimulus interval was set at ≥15 seconds for scotopic 0.01 electroretinography, ≥60 seconds for scotopic 3 and 30 electroretinographies, and at 1.0 second for photopic electroretinographies. Chart & Scope software (AD instruments) was used to analyze the amplitudes of the a-wave, which has been reported to reflect rod function (Hood and Birch, 1990b) and the b-wave, which has been reported to derived from bipolar cells (Hood and Birch, 1996).

### 4.7 Histological evaluation of retinas and optic nerve

Eyeballs of 12-month-old GLAST (+/−) mice, 18-month-old GLAST (+/−) mice, 21-day-old *rd10* mice, 37-day-old *rd10* mice, and 19-month-old *rd12* mice were enucleated after pentobarbital overdose. To identify the superior portion of the retina, a suture was placed on the edge of the superior conjunctiva. The eyes were fixed in 4% paraformaldehyde for 24 hours at 4 °C and embedded in paraffin. Through the suture and at the point of insertion of the optic nerve, serial 6-μm paraffin-embedded sections were cut. Sections including the center of the optic nerve head were stained with hematoxylin and eosin (HE), or with antibodies, and photographed under an optical microscope (BZ-9000; Keyence).

Eyeballs and linked optic nerves of 18-month-old GLAST (+/−) mice were cut and then fixed in 4% paraformaldehyde for 24 hours at 4 °C and embedded in paraffin. Serial 6-μm paraffin-embedded sections were cut to obtain optic nerve cross-sections (Nakano et al., 2016).

### 4.8 Electron microscopy

Eyeballs of 21-, 30-, and 37-day-old *rd10* mice were enucleated after perfusion fixation with 4.0% paraformaldehyde and were immediately postfixed in a mixture of 2.5% glutaraldehyde and 10% formaldehyde overnight. The tissue was further fixed by immersing in 1% osmium tetroxide for 90 minutes. The tissue was then dehydrated (with a graded series of ethanol [50–100%] baths), cleared in propylene oxide, and embedded in epoxy resin. Ultrathin sections were cut with an ultramicrotome and stained with uranyl acetate and lead citrate and then were examined by transmission electron microscopy (H-7650; Hitachi Co.).

### 4.9 Retinal flat mounts

Eyeballs of 12-month-old GLAST (+/−):Thy1-CFP mice were enucleated after pentobarbital overdose and then, after 1 hour fixation in 4% paraformaldehyde, retinal flat mounts without uvea and sclera were made (Nakano et al., 2016). CFP positive retinal ganglion cells were manually counted in a 250-μm square at a distance of 1200 μm away from the disc center on the retinal flat mounts in a masked fashion (Fig. 8A).

### 4.10 Western blotting

Eyeballs of 19-month-old *rd12* mice and 19-, 21-, 23-, 30-, and 37-day-old *rd10* mice were enucleated after pentobarbital overdose, and immediately after enucleation, eyeballs were immersed in cold Hanks' balanced salt solution. Incisions were made using pinholes at the corneas, then using the incisions the sclera was peeled to take the mixture of the retinal pigment epithelium, choroid and sclera separately from the neural retina. The lens and iris were removed, and then separate extracts were prepared from dissected neural retina and the mixture of retinal pigment epithelium, choroid, and sclera (RPE/choroid), and these were analyzed by standard western blotting techniques. Two eyes from two 19-month-old *rd12* mice with supplemental BCAAs and two eyes from two mice without BCAA treatment, and four eyes from four 19-,21-,23-,30-, and 37-day-old *rd10* mice and four eyes from four mice without BCAA treatment were analyzed. The relative intensities of bands were quantified using Image Lab 4.1 (Bio-Rad).

### 4.11 Antibodies

Anti-CHOP and anti-short wavelength sensitive opsin (S-opsin) antibodies were purchased from Santa Cruz Biotechnology; anti-middle wavelength sensitive opsin (M-opsin) and anti-actin antibodies were purchased from Millipore; anti-cleaved caspase-3, anti-phospho-mTOR (Ser 2481), anti-phospho-mTOR (Ser 2448), anti-mTOR, anti-phospho-p70S6K (pp70S6K), anti-phospho-4E-BP1 antibodies were purchased from Cell Signaling Technology, an anti-LC3 antibody was purchased from MBL, an anti-LAMP2 antibody was purchased from Abcam transduction Laboratories, and an anti-p62 (lck ligand) antibody was purchased from BD.

### 4.12 Statistical analysis

Data are presented as mean ± standard deviation. Unpaired *t*-tests were used to compare parameters of mice administered BCAAs or minus-BCAA controls. A Tukey HSD test or Tamhane test was used to compare parameters with multiple conditions in HeLa or 661W cells. The level of statistical significance was set at p < 0.05.

## Declarations

### Author contribution statement

Tomoko Hasegawa: Conceived and designed the experiments; Performed the experiments; Analyzed and interpreted the data; Wrote the paper.

Hanako Ohashi Ikeda: Performed the experiments; Analyzed and interpreted the data; Wrote the paper.

Sachiko Iwai, Yuki Muraoka, Keiko Okamoto-Furuta, Haruyasu Kohda: Performed the experiments.

Tatsuaki Tsuruyama: Performed the experiments; Contributed reagents, materials, analysis tools or data.

Akira Kakizuka: Conceived and designed the experiments; Analyzed and interpreted the data; Wrote the paper.

Nagahisa Yoshimura: Conceived and designed the experiments.

### Funding statement

This work was supported in part by Research grants from the Astellas Foundation for Research on Metabolic Disorders, the Japan Foundation for Applied Enzymology, the Uehara Memorial Foundation, Mochida Memorial Foundation for Medical and Pharmaceutical Research, YOKOYAMA Foundation for Clinical Pharmacology (YRY1308), Japan Intractable Diseases Research Foundation, Japan Research Foundation for Clinical Pharmacology, Kobayashi Magobe memorial medical Foundation, a Grant-in-Aid for Young Scientists (24791850, H.O.I, 16K20316, S.I.), grants from SORST of JST (A.K.), the Ministry of Education, Culture, Sports, Science, and Technology of Japan (A.K., H.O.I. and N.Y.), and the Ministry of Health, Labour and Welfare of Japan (A.K., H.O.I. and N.Y.), and the Innovative Techno-Hub for Integrated Medical Bio-Imaging of the Project for Developing Innovation Systems (N.Y.) from the Ministry of Education, Culture, Sports, Science, and Technology of Japan.

### Competing interest statement

The authors declare the following conflict of interests: in relation to this manuscript, Kyoto University and Ajinomoto Co. applied for patents (PCT/JP2016/063914, 2016-023044), and Tomoko Hasegawa, Hanako Ohashi Ikeda, Yuki Muraoka, Akira Kakizuka, and Nagahisa Yoshimura were inventors of the applied patents. The other authors declare no competing interests. Ajinomoto Co. provided formulations of BCAAs to the authors' institution. Ajinomoto Co. measured the BCAA concentration in eyes.

### Additional information

Supplementary content related to this article has been published online at https://doi.org/10.1016/j.heliyon.2018.e00544.

No additional information is available for this paper.
