# Supplementary material for: Branched chain amino acids attenuate major pathologies in mouse models of retinal degeneration and glaucoma
Source: Heliyon. 2018 Mar 1;4(2):e00544. doi: 10.1016/j.heliyon.2018.e00544 (PMC5857634; doi:10.1016/j.heliyon.2018.e00544)
Supplement: Supplementary Figures [file mmc1.pdf]

Figure S1

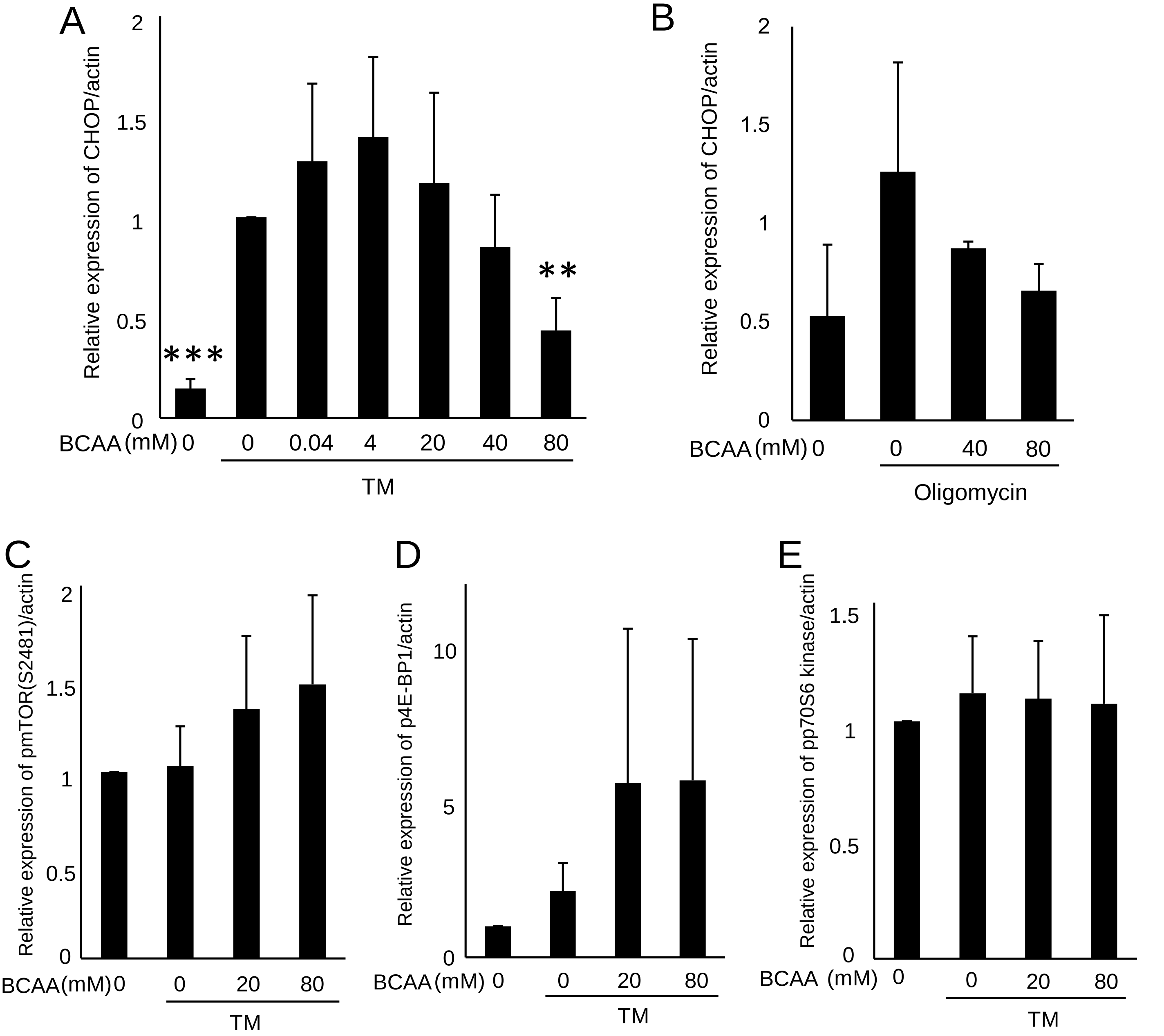
A
Relative expression of CHOP/actin
2
1.5
1
0.5
0
***
**
BCAA (mM) 0
0 0.04 4 20 40 80
TM
B
Relative expression of CHOP/actin
2
1.5
1
0.5
0
BCAA (mM) 0
0 40 80
Oligomycin
C
Relative expression of pmTOR(S2481)/actin
2
1.5
1
0.5
0
BCAA(mM) 0
0 20 80
TM
D
Relative expression of p4E-BP1/actin
10
5
0
BCAA (mM) 0
0 20 80
TM
E
Relative expression of pp70S6 kinase/actin
1.5
1
0.5
0
BCAA (mM) 0
0 20 80
TM

Figure S2

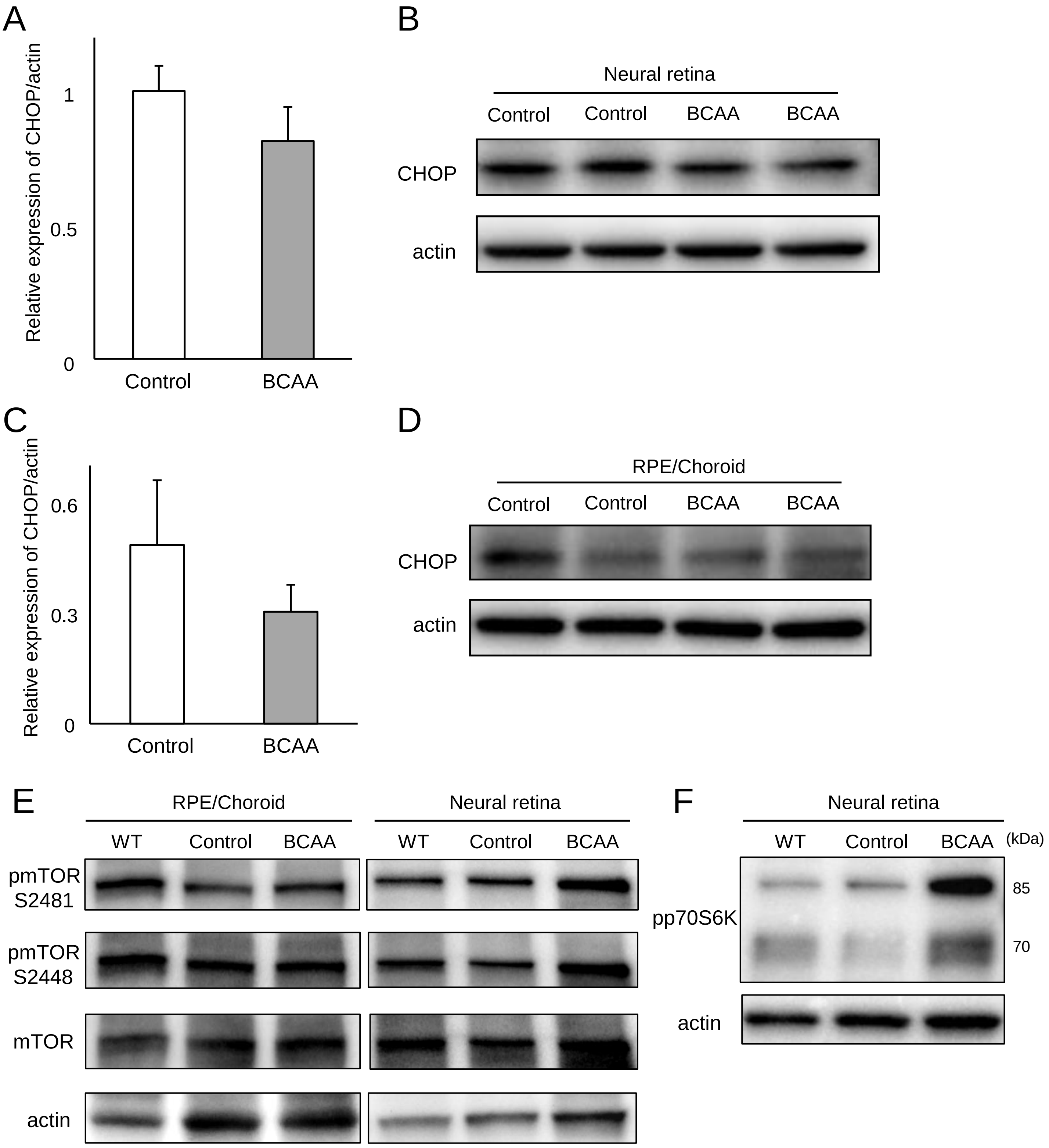
A
Relative expression of CHOP/actin
1
0.5
0
Control
BCAA
B
Neural retina
Control
Control
BCAA
BCAA
CHOP
actin
C
Relative expression of CHOP/actin
0.6
0.3
0
Control
BCAA
D
RPE/Choroid
Control
Control
BCAA
BCAA
CHOP
actin
E
RPE/Choroid
Neural retina
WT
Control
BCAA
WT
Control
BCAA
pmTOR S2481
pmTOR S2448
mTOR
actin
F
Neural retina
WT
Control
BCAA
(kDa)
pp70S6K
85
70
actin

Figure S3

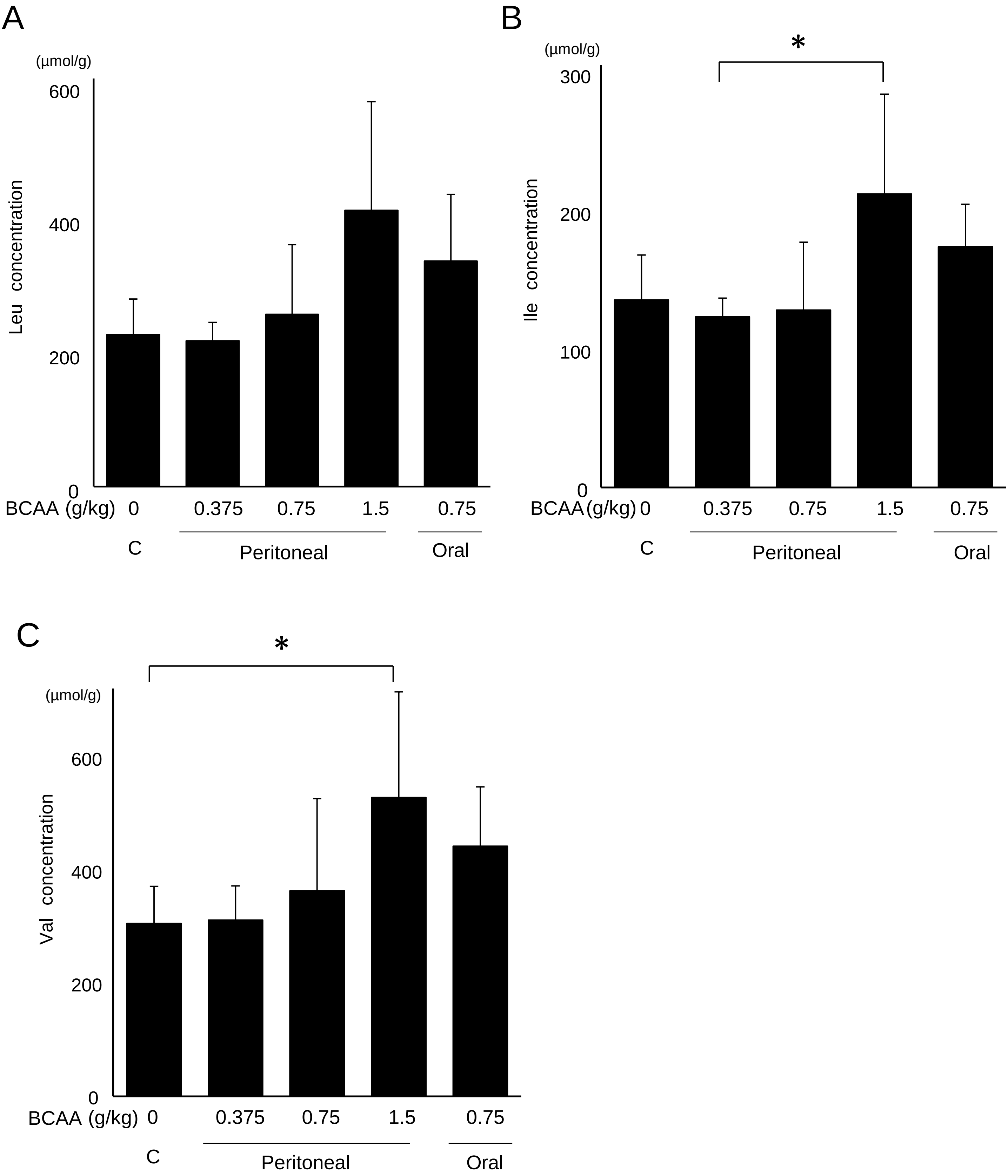
A
(µmol/g)
600
400
200
0
Leu concentration
BCAA (g/kg) 0 0.375 0.75 1.5 0.75
C
Peritoneal
Oral
B
*
(µmol/g)
300
200
100
0
Ile concentration
BCAA (g/kg) 0 0.375 0.75 1.5 0.75
C
Peritoneal
Oral
C
*
(µmol/g)
600
400
200
0
Val concentration
BCAA (g/kg) 0 0.375 0.75 1.5 0.75
C
Peritoneal
Oral

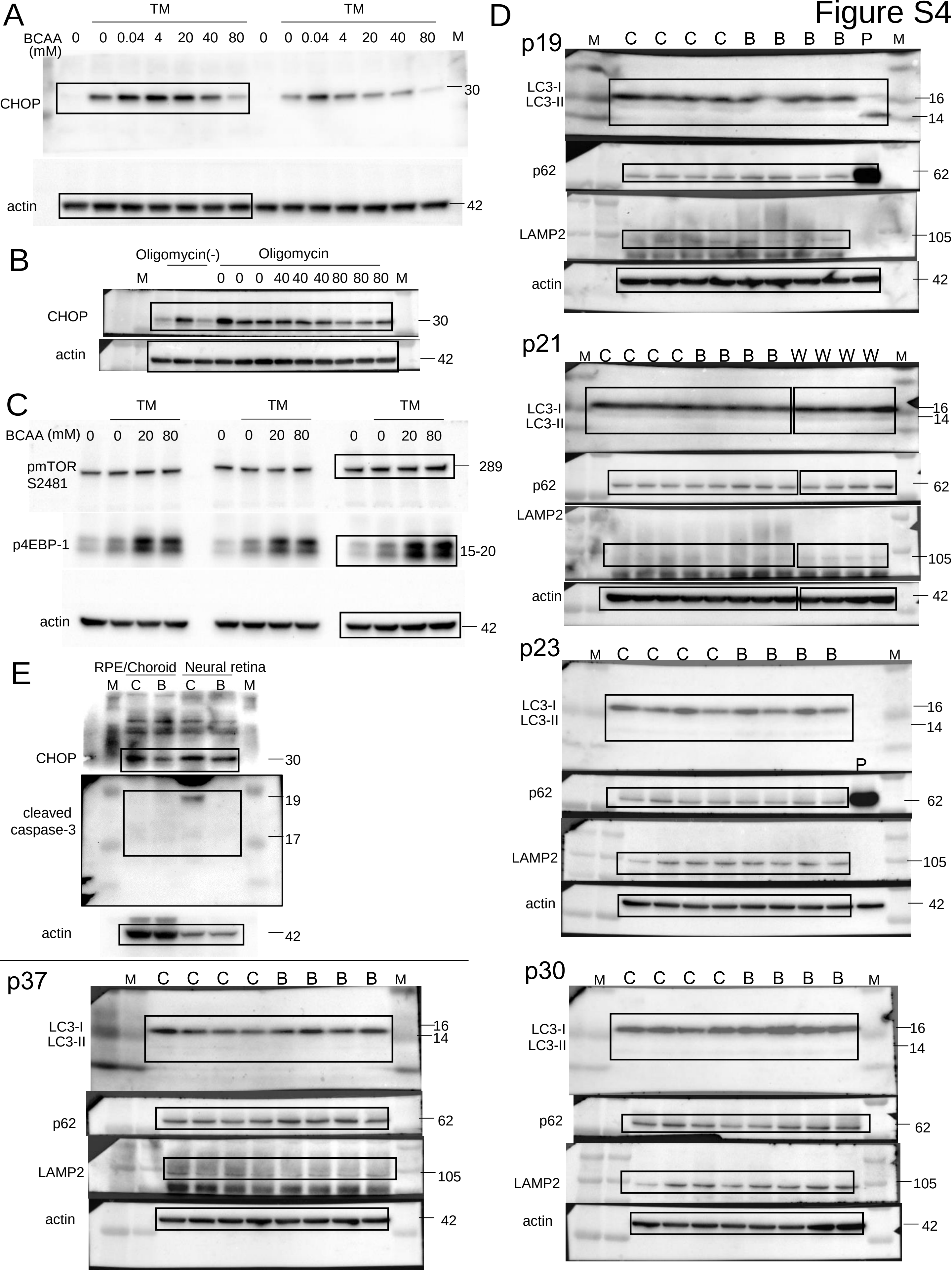
Figure S4
A
TM
TM
BCAA (mM) 0 0 0.04 4 20 40 80 0 0 0.04 4 20 40 80 M
CHOP
30
actin
42
B
Oligomycin(-)
Oligomycin
M 0 0 0 40 40 40 80 80 80 M
CHOP
30
actin
42
C
TM
TM
TM
BCAA (mM) 0 0 20 80 0 0 20 80 0 0 20 80
pmTOR S2481
289
p4EBP-1
15-20
actin
42
D
p19
M C C C C B B B B P M
LC3-I
LC3-II
16
14
p62
62
LAMP2
105
actin
42
p21
M C C C C B B B B W W W W M
LC3-I
LC3-II
16
14
p62
62
LAMP2
105
actin
42
p23
M C C C C B B B B M
LC3-I
LC3-II
16
14
P
p62
62
LAMP2
105
actin
42
p30
M C C C C B B B B M
LC3-I
LC3-II
16
14
p62
62
LAMP2
105
actin
42
E
RPE/Choroid
Neural retina
M C B C B M
CHOP
30
cleaved caspase-3
19
17
actin
42
p37
M C C C C B B B B M
LC3-I
LC3-II
16
14
p62
62
LAMP2
105
actin
42

Figure S5

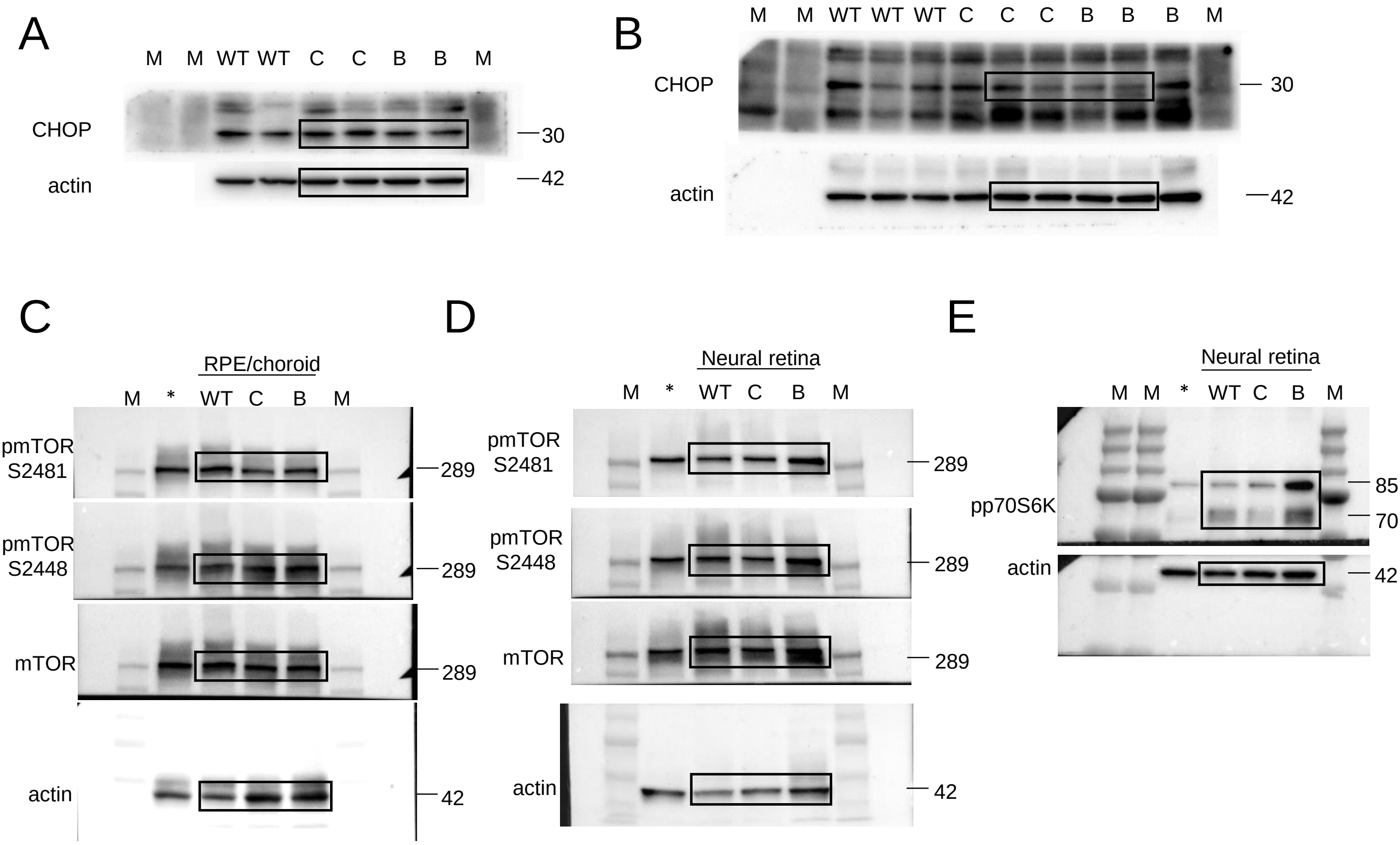
A
M M WT WT C C B B M
CHOP
30
actin
42
B
M M WT WT WT C C C B B B M
CHOP
30
actin
42
C
RPE/choroid
M * WT C B M
pmTOR S2481
289
pmTOR S2448
289
mTOR
289
actin
42
D
Neural retina
M * WT C B M
pmTOR S2481
289
pmTOR S2448
289
mTOR
289
actin
42
E
Neural retina
M M * WT C B M
pp70S6K
85
70
actin
42

Figure S1. Suppression of CHOP and activation of mTOR in cultured cells. Related to Figure 3. (A, C, D, E) HeLa cells were cultured with tunicamycin (TM) (3 μg/mL), with or without branched chain amino acids (BCAA) for 6 hours. (B) 661W cells were cultured with oligomycin (1 μg/mL) with or without BCAAs for 24 hours. CHOP (A and B), phosphorylated mTOR (Ser 2481) (C), phosphorylated 4E-BP1 (D), and phosphorylated p70 S6 kinase (E) were analyzed. The relative intensities of bands were quantified, and the ratios to those of actin were analyzed. $^{**}p < 0.01$ $^{***}p < 0.001$, with the following statistical tests: Tamhane, (A) vs. TM treatment without BCAAs. N = 5 for A, C, D, E, and N = 3 for B.

Figure S2. Suppression of ER stress and activation of mTOR by BCAAs in *rd12* mice. Related to Figure 9. Western blot analysis of 19-month-old *rd12* mice treated with BCAAs, or water as a control. Extracts from dissociated neural retinas (A, B, E and F) and the combination of retinal pigment epithelium (RPE), choroid and sclera (RPE/choroid) (C, D and E) were separately analyzed. (A-D) CHOP, (E) Phosphorylated mammalian target of rapamycin (pmTOR, phosphorylated at S2481 and S2448) and (F) phosphorylated phosphoprotein 70 ribosomal protein S6 kinase (p70S6K) were analyzed. Actin was used as a loading control. Complete scans of western blots are shown in Figures S5A—E. WT: age-matched wild-type mice.

Figure S3. Concentration of BCAAs in eyes increased in a dose-dependent manner. Related to Materials & Methods. BCAA concentrations in eyeballs were measured 2 hours after a single administration of BCAAs (the doses for intraperetonial injection were 0, 0.375, 0.75, or 1.5 g/kg; the dose for oral administration was 0.75 g/kg). Eyeballs of 2-month-old wild-type mice were enucleated after pentobarbital overdose. Immediately after enucleation, eyeballs were weighed and then frozen. Concentration of (A) leucine (Leu), (B) isoleucine (Ile), and (C) valine (Val) were measured at Ajinomoto Co. using a UF-Amino Station system. Key: C, no administration control; Peritoneal, intraperitoneal administration of BCAAs; Oral, oral administration of BCAAs. $^{*}p < 0.05$, Tukey HSD test in B and C.

Figure S4. Complete scans of the western blots. Related to Figures 3, 5, and 9. Complete scans of the western blots in Figure 3A (A), 3B (B), 3C (C), 5 (D), 9 (E).

(A and C) HeLa cells were cultured with tunicamycin (TM) (3 μg/mL) with or without BCAAs for 6 hours. CHOP (A), phosphorylated mTOR (phosphorylated at S2481) and phosphorylated 4EBP-1 (C) were analyzed.

(B) 661W cells were cultured with oligomycin (1 μg/mL) with or without BCAAs for 24 hours. CHOP were analyzed.

(D) Extracts from dissociated neural retinas and the combination of RPE, choroid and sclera (RPE/choroid) from 19-, 21-, 23-, 30- and 37-day-old *rd10*

mice were analyzed with antibodies against p62, LC3 or LAMP2.
(E) Neural retinas and the combination of RPE, choroid and sclera (RPE/choroid,) from 19-month-old *rd12* mice were separately collected and analyzed by western blotting. CHOP, cleaved caspase-3 were analyzed.
Abbreviations: C, control; B, BCAAs; W, wild-type mice; P, positive control; M, molecular weight markers. Actin was used as a loading control.

Figure S5. Complete scans of the western blots. Related to Figure S2.
Neural retinas and the combination of RPE, choroid and sclera (RPE/choroid,) from 19-month-old *rd12* mice were separately collected and analyzed by western blotting. CHOP (A and B), phosphorylated mTOR (phosphorylated at S2481 or S2448) and mTOR (C and D), and phosphorylated p70S6K (E) were analyzed. Abbreviations: C, control; B, BCAAs; M, molecular weight markers. WT, 16-month-old wild-type mice. Actin was used as a loading control.
